# MAE-FMD: Multi-agent evolutionary method for functional module detection in protein-protein interaction networks

**DOI:** 10.1186/1471-2105-15-325

**Published:** 2014-09-30

**Authors:** Jun Zhong Ji, Lang Jiao, Cui Cui Yang, Jia Wei Lv, Ai Dong Zhang

**Affiliations:** College of Computer Science, Beijing University of Technology, Chaoyang District, Beijing, China; Department of Computer Science and Engineering, State University of New York at Buffalo, Buffalo, New York USA

**Keywords:** Computational biology, Protein-protein interaction network, Functional module detection, Multi-agent evolution

## Abstract

**Background:**

Studies of functional modules in a Protein-Protein Interaction (PPI) network contribute greatly to the understanding of biological mechanisms. With the development of computing science, computational approaches have played an important role in detecting functional modules.

**Results:**

We present a new approach using multi-agent evolution for detection of functional modules in PPI networks. The proposed approach consists of two stages: the solution construction for agents in a population and the evolutionary process of computational agents in a lattice environment, where each agent corresponds to a candidate solution to the detection problem of functional modules in a PPI network. First, the approach utilizes a connection-based encoding scheme to model an agent, and employs a random-walk behavior merged topological characteristics with functional information to construct a solution. Next, it applies several evolutionary operators, i.e., competition, crossover, and mutation, to realize information exchange among agents as well as solution evolution. Systematic experiments have been conducted on three benchmark testing sets of yeast networks. Experimental results show that the approach is more effective compared to several other existing algorithms.

**Conclusions:**

The algorithm has the characteristics of outstanding recall, F-measure, sensitivity and accuracy while keeping other competitive performances, so it can be applied to the biological study which requires high accuracy.

## Background

With the completion of the sequencing of the human genome, proteomic research becomes one of the most important areas in the life science [1]. Proteomics is the systematic study of the diverse properties of proteins to provide detailed descriptions of the structure, function and control of biological systems in health and disease [2], where the analysis of underlying relationships in protein data can potentially yield and considerably expand useful insights into roles of proteins in biological processes. That is, protein-protein interactions (PPI) can provide us with a good opportunity to systematically analyze the structure of a large living system and also allow us to use them to understand essential principles. Therefore, the analysis of PPI networks naturally serves as the basis to a better understanding of cellular organization, processes, and functions [3]. Since biologists have found that cellular functions and biochemical events are coordinately carried out by groups of proteins interacting each other in functional modules (or complexes), and the modular structure of a complex network is critical to functions, identifying such functional modules (or complexes) in PPI networks is very important for understanding the structures and functions of these fundamental cellular networks^a^. In the last decade, some biological experimental methods, e.g., tandem affinity purification with mass spectrometry [4, 5] and protein-fragment complementation assay (PCA) [6], have already been used to detect functional modules in PPI networks. However, there are several limitations to these experimental methods, such as too many processing steps and too time-consuming, especially when dealing with a large-scale and densely connected PPI network. Therefore, computational approaches based on machine learning and data mining have been designed and become useful complements to the experimental methods. Over the last decade, a variety of classic clustering approaches, such as density-based clustering [7–9], hierarchical clustering [10–12], partition-based clustering [13–15], and flow simulation-based clustering [16–18], have been used for identifying functional modules in PPI networks. In recent years, there has also been a number of new emerging approaches [19–21], which employs novel computational models to identify functional modules in a PPI network. Especially, some nature-inspired swarm intelligence algorithms have been recently applied to the detection of functional modules in PPI networks [22–25]. Though using computational approaches to detect protein functional modules in PPI networks has received considerable attention and researchers have proposed many detection ideas and schemes over the past few years [1], how to efficiently identify functional modules by means of novel computational approaches is still a vital and challenging scientific problem in computational biology.

Agent-based methods have been previously applied to solving certain search and optimization problems [26, 27]. In such methods, an agent, *a*, is a computational entity that resides in and reacts to its local environment. During the process of interacting with its environment and companion agents, each agent increases its energy level as much as possible, so that the multi-agent evolution can achieve the ultimate goal of solving a global optimization problem. As another example of nature-inspired methods, multi-agent evolution has shown some promises in producing low-cost, fast, and reasonably accurate solutions to certain computational problems, such as classification [28], clustering [29, 30], and social network community mining [31]. These encouraging applications are significant motivation for our research, thus we propose a novel multi-agent evolutionary method to detect functional modules in PPI networks (called MAE-FMD) in this paper. Based on a probability model, MAE-FMD first employs a group of agents as a population to carry out random walks from a start protein to other proteins in a PPI network and finish their individual solution encodings. Then, it randomly places these agents into an evolutionary environment modeled as a lattice, and performs innovative agent-based operations, i.e., competition, cooperation, and mutation, in an attempt to increase the energy levels of agents at each iteration. Experimental results and related comparisons have shown that the MAE-FMD algorithm is effective in achieving better functional module mining results.

## Method

### Basic ideas

In this section, we describe a global search algorithm based on a multi-agent evolutionary method for functional module detection, which consists of two phases: (1) the solution construction phase, and (2) the solution evolution phase. In the first phase, each agent traverses all the nodes of a PPI network through a random-walk process and forms its own solution. In the second phase, the population of agents (i.e., all solutions) are randomly placed into an evolutionary environment for their iterative evolutions until a predefined termination criterion is satisfied. During the evolutions, an energy level is employed to evaluate the ability of an agent to solve a problem in the multi-agent system. The higher the energy level of an agent, the better the quality of the corresponding solution.

### Agent representation and its construction

**Figure 1:**
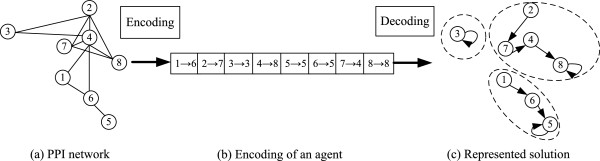
**The connection-based encoding of an agent.** **(a)** PPI network; **(b)** Encoding of an agent; **(c)** Represented solution.

In the MAE-FMD algorithm, each agent corresponds to a candidate solution. An agent is encoded as a graph with *N* directed edges: *A*={(1→*a*_1_),(2→*a*_2_),⋯,(*i*→*a*_*i*_),⋯,(*N*→*a*_*N*_)}, where *i* is a node label, *a*_*i*_ denotes the connected node from *i*^*th*^ node in the represented solution, and *N* is the number of nodes in a PPI network. Take the PPI network shown in Figure 1(a) as an example. It consists of eight nodes numbered from 1 to 8. Figure 1(b) gives an encoding form of its corresponding agent, which can be translated into the graph structure as given in Figure 1(c), where each connected component provides a group of nodes, corresponding to the same partition of the network as shown in Figure 1(a).

To obtain a feasible solution, an agent proceeds from a start node and continuously employs a random-walk behavior to traverse other nodes in a PPI network. At each time step, the agent is on a node, tries to move to a functionally related or similar node that is chosen probabilistically from its topologically adjacent nodes, and builds a corresponding connection. When there is no any satisfied node, the agent will end its current traversal by pointing to itself and then randomly select an untraversed node in a PPI network and begin to a new traversal. This random-walk behavior will be performed until all nodes have been processed. Thereafter, the agent forms its solution. A main advantage of this solution is that the number *K* of clusters is automatically determined by the number of components obtained by an agent, namely, those nodes with a connected relationships are automatically classified into the same community during a later decoding process. Obviously, such an encoding method does not rely on knowing number of clusters beforehand.

During the random-walk process, an agent constructs a solution by proceeding from a start node and moving to feasible neighborhood nodes in a step-by-step fashion. In each step, an agent *k* moves from node *i* to node *j* based on the following probability:
1

where *s*_*i*,*j*_ denotes a measure of connection strength between two nodes *i* and *j* from the view of topology structures, *f*_*i*,*j*_ is a functional similarity score of the two nodes *i* and *j*, and  is a set of available nodes in which each one *l* (or *j*) is a neighborhood node of node *i* not yet visited by the *k*^*th*^ agent in the current traversal and (*s*_*i*,*j*_+*f*_*i*,*j*_)≥*ε* (*ε* represents a specified strength threshold for the combination of topology and function similarities).

Given two nodes *i*,*j*∈*V*, we compute their connection strength by using the structural similarity formula as follows [32]:
2

where *Γ*(*i*) is a set of the neighborhood nodes of node *i*, and |*Γ*(*i*)| is the size of the set.

Based on the annotation information of Gene Ontology (GO), the functional similarity measure for proteins can be implemented. For two proteins *i* and *j* that are annotated with two GO term sets *g*^*i*^ and *g*^*j*^, respectively, the functional similarity score can be calculated by [33]:
3

### Agent energy level and evolutionary environment

According to the meaning of energy level mentioned above, we are interested in searching a graph partition with the largest energy level. To guarantee highly intra-connected and sparsely inter-connected modules, we adopt the modularity density function [34] to compute the energy level of an agent:
4

where *K* is the number of detected modules for an agent *A*, *e*_*c*_ is the number of links between nodes in *c*_*th*_ module, |*E*| is the number of all links in the PPI network, and *d*_*c*_ is the sum of the degrees of nodes in *c*_*th*_ module. During each evolutionary process, an agent will try to increase its energy level as much as possible by sensing and performing some reactive behaviors to survive.

**Figure 2:**
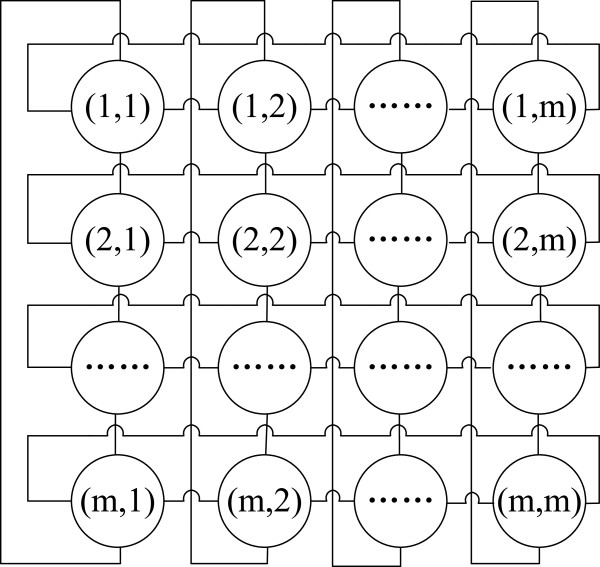
**The lattice environment for the agent evolutionary.**

To realize the local perceptivity of agents, we select the common lattice structure used in [27, 29, 30] as the evolutionary environment, which is more close to the real evolutionary mechanism in nature than the model of the population in traditional Genetic Algorithms (GAs). All *M* agents in a population live in such a lattice environment. The size of lattices is *m*×*m*, where *m* is an integer and . Each agent is randomly placed on a lattice-point and it can only interact with its neighbors. The agent lattice can be shown as the one in Figure 2. Each agent, who corresponds to a partition solution, can occupy a circle in the evolutionary environment, where the data in a circle represents its position in the lattice structure, and two agents can interact with each other if and only if there is a line connecting them.

Suppose that the agent located at (*u*,*v*) is *A*_*u*,*v*_, *u*,*v*=1,2,…,*m*, then the neighborhood agents of *A*_*u*,*v*_, *Neighbor*(*A*_*u*,*v*_), are defined as follows:
5

where *u*^′^=*mod*(*u*−1+*m*−1,*m*)+1, *v*^′^=*mod*(*v*−1+*m*−1,*m*)+1, *u*^″^=*mod*(*u*,*m*)+1, *v*^″^=*mod*(*v*,*m*)+1.

### Evolutionary operators

In the above evolutionary environment, computational agents will compete or cooperate with others so that they can gain higher energy level. To simulate the evolution phenomenon in a more natural way, each agent can only sense its local environment, and its behaviors of competition and cooperation can only take place between the agent and its neighborhood agents. That is, an agent interacts with its neighborhood agents, and useful information is transferred among them. In such a way, the information can be gradually diffused to the whole lattice environment so that the global evolution of the agent population is realized. To achieve this purpose, three basic operators are designed for detecting communities in a PPI network.

**1) Competition operator.** Suppose that the operator is performed on the agent located at (*u*,*v*), *A*_*u*,*v*_=((1→*a*_1_),(2→*a*_2_),…,(N→*a*_*N*_)), and *H*_*u*,*v*_=((1→*h*_1_),(2→*h*_2_),…,(N→*h*_*N*_)) is another agent with the highest energy level among the neighborhood agents of *A*_*u*,*v*_, namely, *H*_*u*,*v*_∈*Neighbor*(*A*_*u*,*v*_) and ∀*A*^′^∈*Neighbor*(*A*_*u*,*v*_), then *Energy*(*A*^′^)≤*Energy*(*H*_*u*,*v*_). If *Energy*(*A*_*u*,*v*_)≥*Energy*(*H*_*u*,*v*_), *A*_*u*,*v*_ is a winner, so it can still live in the original lattice; otherwise it will die as a loser, and its lattice-point will be occupied by *H*_*u*,*v*_. *H*_*u*,*v*_ has two candidate strategies to occupy a lattice-point, and it randomly selects one of them with a probability *p*_*o*_. Let *r*(0,1) be a uniform random number generator, the value range of which belongs to (0,1). If *r*(0,1)<*p*_*o*_, occupying strategy 1 is selected; otherwise occupying strategy 2 is carried out. In the two occupying strategies, *H*_*u*,*v*_ first generates its clone agent *C*_*u*,*v*_ = ((1 →*c*_1_), (2 →*c*_2_), …, (N →*c*_*N*_)), and then *C*_*u*,*v*_ is placed on the lattice-point to be occupied.

Let  and , namely, the connection strengths of *A*_*u*,*v*_ are *Al*_1_,*Al*_2_,…,*Al*_*N*_, and the connection strengths of *H*_*u*,*v*_ are *Hl*_1_,*Hl*_2_,…,*Hl*_*N*_, respectively. If a node has no other nodes to be pointed in addition to point to its own, then we call it a breakpoint. In fact, a breakpoint represents the segmentation of two different modules in a PPI network with N directed edges. To distinguish breakpoints, we set  only when *i*=*a*_*i*_ in an agent encoding.

**Strategy 1.** For the connection with the lowest strength in *H*_*u*,*v*_, *Hl*_*j*_=*Min*(*Hl*_1_,*Hl*_2_,…,*Hl*_*N*_), if *Al*_*j*_>*Hl*_*j*_ then *c*_*j*_ is replaced with *a*_*j*_ (*j*=1,2,…,*N*) in the new agent.

**Strategy 2.** Each *Al*_*i*_ of *A*_*u*,*v*_ is respectively compared with the corresponding *Hl*_*i*_ of *H*_*u*,*v*_. If *Al*_*i*_>*Hl*_*i*_, then *c*_*i*_=*a*_*i*_ in the new agent.

**Figure 3:**
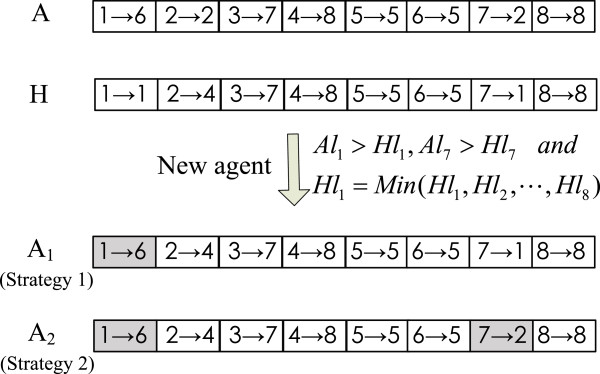
**Competition operator.**

In the following, we take a PPI network with 8 nodes as an example to illustrate these operators. A schematic diagram of a competition operator is given in Figure 3, where *A* = ((1→ 6),(2→ 2),(3→ 7),(4→ 8),(5→ 5),(6→ 5),(7→2),(8→8)) is an agent to participate in a competition, *H*=((1→1),(2→4),(3→7),(4→8),(5→5),(6→5),(7→1),(8→8)) is its neighborhood agent with the highest energy level and *Energy*(*H*)≥*Energy*(*A*), and *A*_1_ and *A*_2_ are two new agents produced by the competition operator where a shape represents a change in the encoding of the clone agent of *H*. Assumpting that *Al*_1_>*Hl*_1_,*Al*_7_>*Hl*_7_ and *Hl*_1_=*Min*(*Hl*_1_,*Hl*_2_,···,*Hl*_8_), *A*_1_ is the result of Strategy 1 where the link (1→1) is replaced with (1→6) while *A*_2_ is that of Strategy 2 where the two links (1→1) and (7→1) are respectively replaced with (1→6) and (7→2).

In fact, the two strategies in this operator are designed to play similar roles. More specifically, Strategy 1 only replaces the worst connection of a winner with the better information of a loser while Strategy 2 is in favor of reserving all advantaged information of a loser.

**2) Crossover operator.** Suppose that two parent agents are *F*_1_=((1→*f*_1_),(2→*f*_2_),…,(N→*f*_*N*_)) and *F*_2_=((1→*f*1′),(2→*f*2′),…,(N→*fN*′)) which will randomly produce a child agent *C*_1_=((1→*c*_1_),(2→*c*_2_),…,(N→*c*_*N*_)) by making use of their connection information and the corresponding crossover strategies. To obtain offsprings of the two parent agents, the rules of crossover operator are as follows.

**Alternating link crossover rule.** The rule works as follows: first it chooses a link from the first parent at random; secondly, the link is extended with the appropriate link of the second parent; thirdly, the partial tour created in this way is extended with the appropriate link of the first parent, etc. This process is repeated until traversing all the nodes in a PPI network. During the generation of a candidate agent, once a link is chosen which would produce a cycle into the partial tour, the next link will be selected randomly from the links of those untraversed nodes in the corresponding parent.

**Figure 4:**
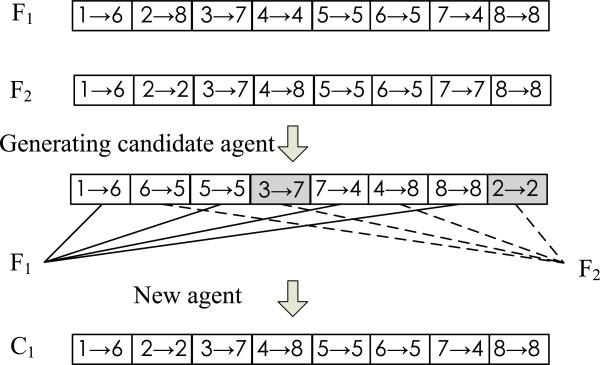
**Alternating link crossover operator.**

The schematic diagram of the alternating link crossover rule is shown in Figure 4, where *F*_1_=((1→6),(2→8),(3→7),(4→4),(5→5),(6→5),(7→4),(8→8)) and *F*_2_=((1→6),(2→2),(3→7),(4→8),(5→5),(6→5),(7→7),(8→8)) are two parent agents, and *C*_1_=((1→6),(2→2),(3→7),(4→8),(5→5),(6→5),(7→4),(8→8)) is an offspring agent. In generating a candidate agent, the links (1→6),(5→5),(7→4) and (8→8) are selected from the first parent while the other links from the second parent, and each shape represents a starting point of a new subtour in the candidate agent.

**Alternating chunk crossover rule.** Based on this rule, an offspring is constructed from two parent agents as follows: first it takes a random length subtour of the first parent; then this partial tour is extended by choosing a subtour of random length from the second parent; next the partial tour is constantly extended by taking subtours from alternating parents till up to the length of the solution. For each subtour, the random length range is 1 to the remaining digits of the constructing solution. In generating a candidate agent, if a link is chosen which would produce a cycle into the partial tour, the next link will be selected randomly from the links of those untraversed nodes in the corresponding parent. Different length subtours from two parent agents are alternatingly chosen to construct a child agent.

**Figure 5:**
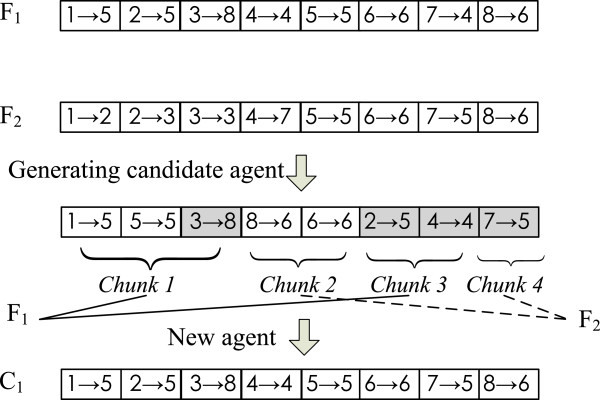
**Alternating chunk crossover operator.**

Figure 5 gives an illustrative diagram of an alternating chunk crossover rule, where *F*_1_=((1→5),(2→5),(3→8),(4→4),(5→5),(6→6),(7→4),(8→6)) and *F*_2_=((1→2),(2→3),(3→3),(4→7),(5→5),(6→6),(7→5),(8→6)) are two parent agents, and *C*_1_=((1→5),(2→5),(3→8),(4→4),(5→5),(6→6),(7→5),(8→6)) is an offspring agent. In generating a candidate agent, we assume that the sizes of four chunks are respectively determined as 3, 2, 2 and 1 by four random functions, and chunk 1 and chunk 3 are selected from the first parent while the other two chunks from the second parent. The new agent is alternatively constructed by means of the subtours of different parents, where shapes represent the same meaning as in Figure 4.

Obviously, the crossover operator has the function of a random search, which is performed on an agent and its neighborhood agents to achieve the purpose of cooperation with a crossover probability *p*_*c*_. More specifically, if *r*(0,1)<*p*_*c*_, then the algorithm performs the two crossover operators. Otherwise, it skips these operators. Once a child agent has higher energy level than its parent agent after performing crossover operators, the initial agent with lower energy level will be replaced with the child agent.

**3) Self-adaptive mutation operator.** In addition to the behaviors of competition and cooperation, an agent can also increase its energy level by using a self-adaptive mutation operator, which depends on the degree of its evolution and controls the number of digits to be mutated. The mechanism of the self-adaptive mutation operator is denoted as:
6

where *n* is the number of mutation digits, *l*_*i*_ is the number of continued stagnation steps for *i*_*th*_ agent, and *r* is the maximum step length at which an agent might have the same energy level. It is not difficult to find that *n* is not only associated with the encoding length of an agent (network size), but also related to the evolutionary process of the agent. More specifically, the larger the network scale, the more the number of potential mutations. On the other hand, the longer the stagnating time of an agent evolution, the more the number of potential mutations.

Based on a mutation probability *p*_*m*_, *n* connection elements of an agent *A* = ((1 → *a*_1_),(2 → *a*_2_),…,(N → *a*_*N*_)) are randomly selected when *r*(0,1)<*p*_*m*_, and then they are mutated by replacing with other nodes possibly connected in the corresponding module.

**Figure 6:**
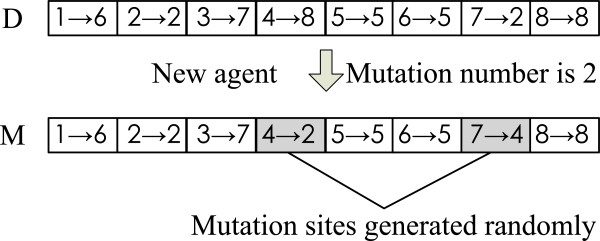
**Self-adaptive mutation operator.**

Figure 6 gives an illustration diagram of a mutation operator, where *D* = ((1→6),(2→2),(3→7),(4→8),(5→5),(6→5),(7→2),(8→8)) is an original agent, its mutation number *n*= 2, *M*= ((1→6),(2→2),(3→7),(4→2),(5→5),(6→5),(7→4),(8→8)) is the mutated agent in which two elements are replaced randomly, and shapes represent the changes in the encoding of the new agent. Essentially, the mutation operator realizes a local search, which only performs a small perturbation on some elements (node connections) of an agent encoding. If a mutation operator can increase the energy level of the current agent, the initial agent with lower energy level will be replaced with the new agent.

In the light of an energy level function, MAE-FMD algorithm employs competition, crossover and mutation operators to continually realize good information exchange among agents and improve the energy levels of a group of initial agents. During the competition process, if the current agent is winner, then it will be kept alive. Otherwise, the neighborhood agent with the highest energy level will be selected, and improved by combination with advantaged information of the current agent, then it replaces the current agent. Meantime, whether crossover operators or mutation operators, once they can produce new agents with higher energy level, the initial agents with lower energy level will be replaced with the new agents. By means of the three operators, the evolutionary process will gradually converge to a solution with the largest energy level which corresponds to the initial module structure of the PPI network.

### Post-processing

After a number of iterations, we can obtain a solution with the largest energy level. That is, the preliminary modules are generated by the multi-agent evolutionary method. To improve the detection quality, we adopt two post-processing strategies based on topological and functional information to produce final modules. The first step is merging the similar preliminary modules in light of functional annotation information. A merging module results from two or more preliminary modules which are close in view of function. The similarity *S*(*M*_*S*_,*M*_*T*_) between two modules *M*_*S*_ and *M*_*T*_ is measured by the functional similarity score defined as:
7

where
8

Two modules with the highest similarity are iteratively merged until there are no such two modules whose similarity is larger than the merging threshold *λ*.

To exclude some too sparsely connected nodes and very small clusters generated above, we perform the filtering step based on the topological density of PPI network subgraphs. The density of subgraphs of functional modules is measured by:
9

where *n*_*s*_ is the number of nodes and *e*_*s*_ is the number of interactions in a subgraph *s* of a PPI network. Let *δ* be a threshold value, those clusters with *D*_*s*_<*δ* and |*s*|<2 will be filtered from clusters generated above. By such two post-processing strategies, the preliminary modules are refined from the topological property and functional similarity, and the potential functional modules hidden in the PPI networks are generated.

### Algorithm description and complexity analysis

The procedure of the proposed MAE-FMD algorithm is to carry out initialization, agent random-walk and solution construction, multi-agent evolution, post-processing, and output of detected modules. The detailed pseudocode is shown in Algorithm 1.

Based on the description of Algorithm 1, the complexity of MAE-FMD can be simply analyzed as follows: Let the maximum number of a node degree be *n*_1_ in a PPI network, and the maximum number of nodes be *n*_2_ in a module. In the initialization process, computing connection strengths (similarities) and the number of common neighbors for all pairs of nodes is time-consuming. For each node, since the number of its maximum neighborhood nodes is *n*_1_, the computing complexity of its all available connection strengths is *n*_1_, thus the time complexity is *O*(*n*_1_·*N*). In the agent random-walk and solution construction process, the time complexity is *O*(*M*·*N*·*n*_1_). In the multi-agent evolution process, the time complexity is . Generally speaking, *K*·*n*_2_≥*N*,*however*,*O*(*K*·*n*_2_)≈*O*(*N*). Thus, the time complexity of the multi-agent evolution process can be simplified as *O*(*T*·(*n*_2_+*M*)·*N*). In the post-processing and output process, the time complexity is *O*(*K*^2^+*K*))≈*O*(*K*^2^). Thus, the overall complexity of MAE-FMD is about *O*(*n*_1_·*N*)+*O*(*M*·*N*·*n*_1_)+*O*(*T*·(*n*_2_+*M*)·*N*)+*O*(*K*^2^). Because most PPI networks are small-world and scale-free networks, *n*_1_≪*N*,*n*_2_<*N*,*K*≪*N*. Moreover, we usually select a constant (e.g, 100) as the population size of agents, which is far less than the number of nodes in a large-scale PPI network. Therefore, the time complexity of MAE-FMD can be decreased to *O*(*T*·(*n*_2_+*M*)·*N*) (*n*_2_<*N* for all PPI networks, *M*≪*N* for a large-scale PPI network), which is better than that of most existing typical algorithms with *O*(*N*^2^). Especially for a large-scale complex network with near uniform community size, the efficiency of MAE-FMD is very promising for detecting modules in PPI networks.


## Results and discussion

In this section, we use three different protein-protein interaction datasets to perform our empirical study. In light of many evaluation metrics, we assess the performance of our algorithm, and compare our test results to other existing algorithms on these PPI datasets. The experimental platform is a PC with Core 2, 2.13 GHz CPU, 2.99 GB RAM, and Windows XP, and all algorithms are implemented by Java language.

### PPI datasets

We have performed our experiments over five publicly available benchmark PPI datasets including four yeast data and one human data, namely DIP data [35], Gavin data [36], MIPS data [37], DIP Scere20140703 and DIP Hsapi20140703. Table 1 shows a summary of the data sets used in our experiments, where the 2th column gives the web links, the 3th and 4th columns respectively present the size of proteins and interactions in source data while the 5th and 6th columns respectively present the size of proteins and interactions in the preprocessed data. A cleaning step, which deletes all self-connected and repeated interactions, is performed in data preprocessing. To evaluate the protein modules mined by our algorithm, the set of real functional modules from [38] is selected as the benchmark. This benchmark set, which consists of 428 protein functional modules, is constructed from three main sources: the MIPS [27], Aloy et al. [39] and the SGD database [40] based on the Gene Ontology (GO) notations.

**Table 1:** **Data sets used in our experiments**

Date sets	Http address	Source data		Preprocessed data	
		Size of P.	Size of I.	Size of P.	Size of I.
Gavin	http://www.thebiogrid.org/[BioGRID version 2.0.33]	1430	6531	1430	6531
DIP	http://dip.doe-mbi.ucla.edu/[version ScereCR20060402]	2554	5952	2528	5728
MIPS	ftp://ftpmips.gsf.de/yeast/PPI/[version PPI18052006]	4554	15456	4545	12318
DIPScere20140703	http://dip.doe-mbi.ucla.edu/dip/Download.cgi?SM=7&TX=4932	5137	22775	5126	22402
DIPHsapi20140703	http://dip.doe-mbi.ucla.edu/dip/Download.cgi?SM=7&TX=9606	4187	6245	4086	5823

### Evaluation metrics

At present, there exist three popular measurements for the evaluation of the detection modules’ quality and the calculation of the detection methods’ general performance [41].

#### Precision, Recall, F-measure, and Coverage

Many research works use a neighborhood affinity score to assess the degree of matching between the identified functional modules and real ones. The score *NA*(*p*,*b*) between an identified module *p*=(*V*_*p*_,*E*_*p*_) and a real module *b*=(*V*_*b*_,*E*_*b*_) in the benchmark module set is defined as:
10

If *NA*(*p*,*b*)≥*ω*, then *p* and *b* are considered to be matched (generally, *ω*=0.2). Let *P* be the set of functional modules identified by some computational methods and *B* be the real functional module set in benchmark networks. And then the number of the modules in *P* which at least matches one real module is denoted by *N*_*cp*_=|{*p*|*p*∈*P*,∃*b*∈*B*,*NA*(*p*,*b*)≥*ω*}|, while the counterpart number in *B* can be denoted by *N*_*cb*_=|{*b*|*b*∈*B*,∃*p*∈*P*,*NA*(*p*,*b*)≥*ω*}|. Thus, Precision and Recall can be defined as follows [42]:
11

and
12

F-measure is a harmonic mean of Precision and Recall, so can be used to evaluate the overall performance. It is defined as:
13

Moreover, Coverage assesses how many proteins in a PPI network can be clustered into the detected modules by a computational method. That is, it indicates the percentage of proteins assigned to any functional module, i.e., 1-Discard-rate, which can be defined as follows [43]:
14

where |*V*|=*N* denotes the size of the PPI network and *V*_*pi*_ is the set of the proteins in the *i*^*th*^ detected module.

#### Sensitivity, positive predictive value, and accuracy

Sensitivity (*S*_*n*_), Positive predictive value (*PPV*) and Accuracy (*Acc*) are also common measures to assess the performance of module detection methods. Let *T*_*ij*_ be the number of the common proteins in both of the *i*^*th*^ benchmark and the *j*^*th*^ identified module. Then *S*_*n*_ and *PPV* can be defined as [38]:
15

and
16

where *N*_*i*_ is the number of the proteins in the *i*^*th*^ benchmark module, and . Generally speaking, *S*_*n*_ assesses how many proteins in the real functional modules can be covered by the predicted modules, while *PPV* indicates that identified modules are more likely to be true positives.

As a general metric, the accuracy of an identification (*Acc*) can be calculated as the geometric mean of *S*_*n*_ and *PPV*:
17

#### p-value measure

Modules can be statistically evaluated using the *p*-value from the hypergeometric distribution, which is defined as [44]:
18

where |*V*| denotes the same means as mentioned in Equation 16, *C* is an identified module, |*F*| is the number of proteins in a reference function, and k is the number of proteins in common between the function and the module. *P*-value is also known as a metric of functional homogeneity. It is understood as the probability that at least k proteins in a module of size |*C*| are included in a reference function of size |*F*|. A low value of p indicates that the module closely corresponds to the function, because it is less probable that the network will produce the module by chance. Consequently, the minimum *p*-value in all modules will show the general performance of each detection method.

### Effects of parameters

In this subsection, we take the Gavin data as an example to study respectively the effects of the algorithm parameters involved in the multi-agent evolution and post-processing. These parameters include the number of agent population (M), the strength threshold of connections (*ε*), the maximum step length with same energy level (*R*), the selection probability (*p*_*o*_), the crossover probability (*p*_*c*_), the mutation probability (*p*_*m*_), merging threshold (*λ*), and filtering threshold value (*δ*). During all experimentations, the value of a single parameter is changed, while keeping the values of other parameters fixed.

**Figure 7:**
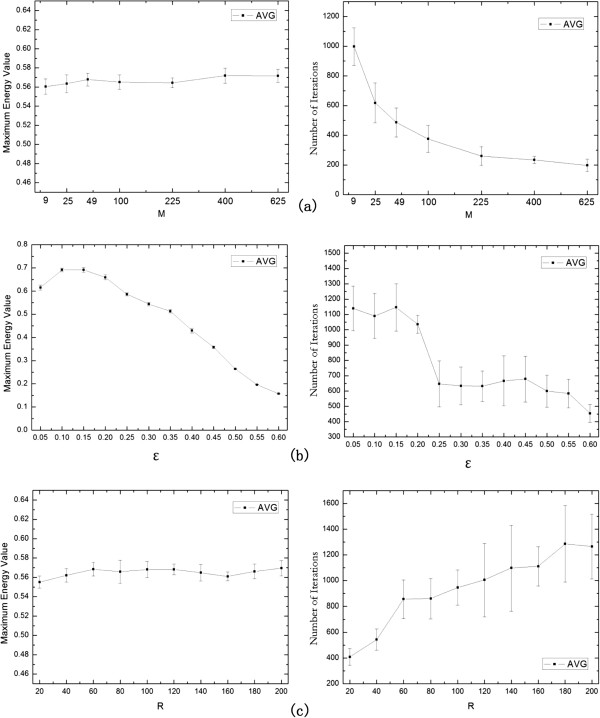
**The effects of three parameters (** ***M*** **,** ***ε*** **,** ***R*** **) on the multi-agent method performance.** **(a)** the plots of the maximum energy value and the number of iterations for different values *M*; **(b)** the plots of the maximum energy value and the number of iterations for different values *ε*; and **(c)** the plots of the maximum energy value and the number of iterations for different values *R*.

For the multi-agent random-walk and evolutionary processes, we take maximum energy of an agent and the number of iterations as two evaluation metrics to test the performance of the algorithm. Ten executions are independently carried out in each parametric combination. Figure 7 reveals that the effects of three main parameters (*M*, *ε*, *R*) on the multi-agent method performance by mean value curve with error bars. Figure 7(a) shows the evolutionary performance with 7 different agent sizes (*M*). Multi-agent evolutionary method is a population-based optimization algorithm, where the number of agent population determines the number of solutions at each iteration. The left graph in Figure 7(a) shows the results about the maximum energy value, and the right graph in Figure 7(a) illustrates the results about the number of iterations. As reflected in Figure 7(a), smaller maximum energy values and larger number of iterationss are obtained when using a small number of agents. Along with the number of agents increasing, the maximum energy slowly increases and the number of iterations decreases on the whole. The reason is that more agents means more initial search points in the search space to be employed so that the search range is larger at each iteration, which induces the algorithm to rapid converge. However, after a sufficient value for the number of agents, any increment does not obviously improve the maximum energy, and also does not dramatically reduce the number of iterations. On the contrary, the search time in each iteration will increase as the size of the number of agents increases. Therefore, to acquire a balance between getting a better solution and using less time, we recommend an agent size of 225 (*M*=225).

The strength threshold of connections *ε* is an important parameter in the constructing solution process of an agent, which controls the feasible neighborhood for each node in an agent random-walk process. To investigate the effect of *ε* on our algorithm, we perform experiments using different values of *ε*. The results are presented in Figure 7(b) where the curve of maximum energy has 2 distinct ranges for various values of *ε*, i.e. [0.05, 0.10] and [0.15, 0.60], while the curve of the number of iterations also has 2 rough ranges for various values of *ε*, i.e. [0.05, 0.20] and [0.25, 0.6]. As *ε* increases, the maximum energy increases in the first range, and then decreases dramatically in the second range. However, our algorithm can keep the maximum energy value being larger than 0.5 when *ε* locates in [0.05, 0.35]. For the curve of the number of iterations, the first range has far larger values than the second range though there are some small fluctuations in both ranges. The reason is as follows: Smaller *ε* is, larger the feasible neighborhood of each node and the search space of a solution are, thus the algorithm will cost more iterations to search a better solution, and vice versa. It is worth noting that the algorithm is easy to fall into local optimal if *ε* is too large though it has s fast convergence performance. Combining above experimental results and such analysis, we select *ε*=0.25 in our algorithm.

The maximum step length with same energy level *R* is also a key parameter which plays an important role in determining the end of evolution. Figure 7(c) shows two plots of the maximum energy value and number of iterations for different *R*. From the left graph in Figure 7(c), the maximum energy value is insensitive to the parameter *R* and increases slightly along with increasing of *R*. From the right graph in Figure 7(c), the number of iterations will have a more significant increase as the parameter *R* increases. These results illustrate that if the algorithm uses a large value of *R*, which is bound to increase the number of iterations and not necessarily able to get a better result. Considering the two factors together, we set *R*=60.

**Figure 8:**
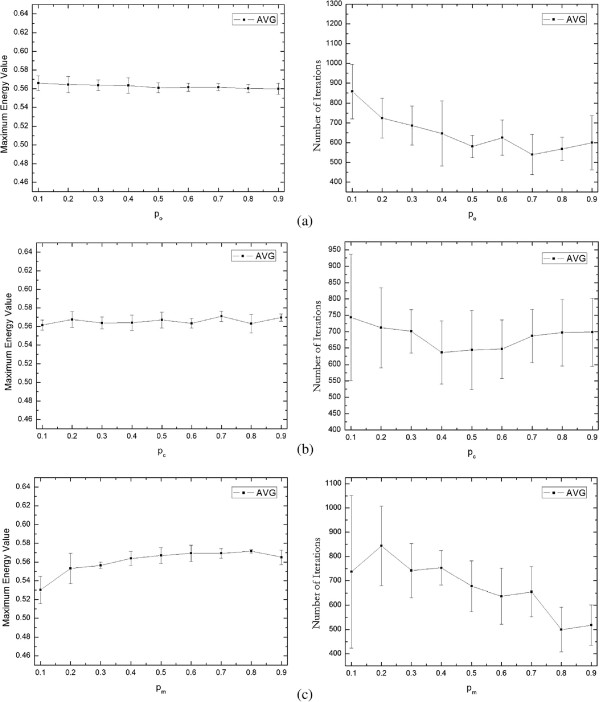
**The effects of three operation parameters** ***(p***_***c***_ ***,p***_***o***_ ***,p***_***m***_ ***)*** **on the multi-agent method performance.** **(a)** indicates how the maximum energy and the iteration number changes when the *p*_*c*_ increases; **(b)** indicates how the maximum energy and the number of iterations changes when the *p*_*o*_ increases; **(c)** indicates how the maximum energy and the iteration number changes when the *p*_*m*_ increases.

Figure 8 reveals that the effects of three operator parameters (*p*_*o*_, *p*_*c*_, *p*_*m*_) on the performance of the multi-agent method by mean value curve with error bars. As shown in the left graph of Figure 8(a), the maximum energy is insensitive to the occupying probability *p*_*o*_, and its value maintains around at 0.56 within all values of *p*_*o*_. From the right graph in Figure 8(a), we can see that the number of iterations decreases as *p*_*o*_ increases on the whole. This is because no matter what value *p*_*o*_ is, the competition operator (Strategy 1 or Strategy 2) is performed, thus the difference on the maximum energy is very small (e.g. the maximum gap of the average value is 0.006). However, since Strategy 2 reserves more advantaged information of a loser than Strategy 1, excessively using Strategy 2 will slow the convergence of the algorithm. Hence, we set *p*_*o*_=0.5 in our algorithm to obtain a balance between two strategies. The relationship curve between the method performance and *p*_*c*_ is shown in the Figure 8(b). Similar to the curve of *p*_*o*_, the maximum energy values vary from the different values of *p*_*c*_, but the difference is very small (e.g. the maximum gap of the average value is 0.009), which suggests that the multi-agent evolutionary process is also not sensitive to the crossover probability. There are three varying ranges for the number of iterations along with *p*_*c*_ increasing, i.e., [0.1, 0.4), [0.4, 0.6] and (0.6, 0.9]. The number of iterations curve decreases gradually in [0.1, 0.4), then maintains the smaller value of almost equal in [0.4, 0.6], and finally increases gradually in (0.6, 0.9], which means that moderate crossover operations can contribute to the convergence of the algorithm, however, too few or too many crossover operations will reduce the convergence of the algorithm. To shorten the evolutionary process, we set *p*_*c*_=0.5 in our algorithm. Figure 8(c) gives the curve of the evolutionary performance on mutation probability *p*_*m*_. As *p*_*m*_ increases, the maximum energy values slowly increase, and the number of iterations decreases with a small amount of fluctuation. That is, the rich mutation operators will not only benefit the convergence of the multi-agent evolutionary process, but also get a better maximum energy value. To obtain a good result and save evolution time, we select *p*_*m*_=0.8 in our algorithm.

**Figure 9:**
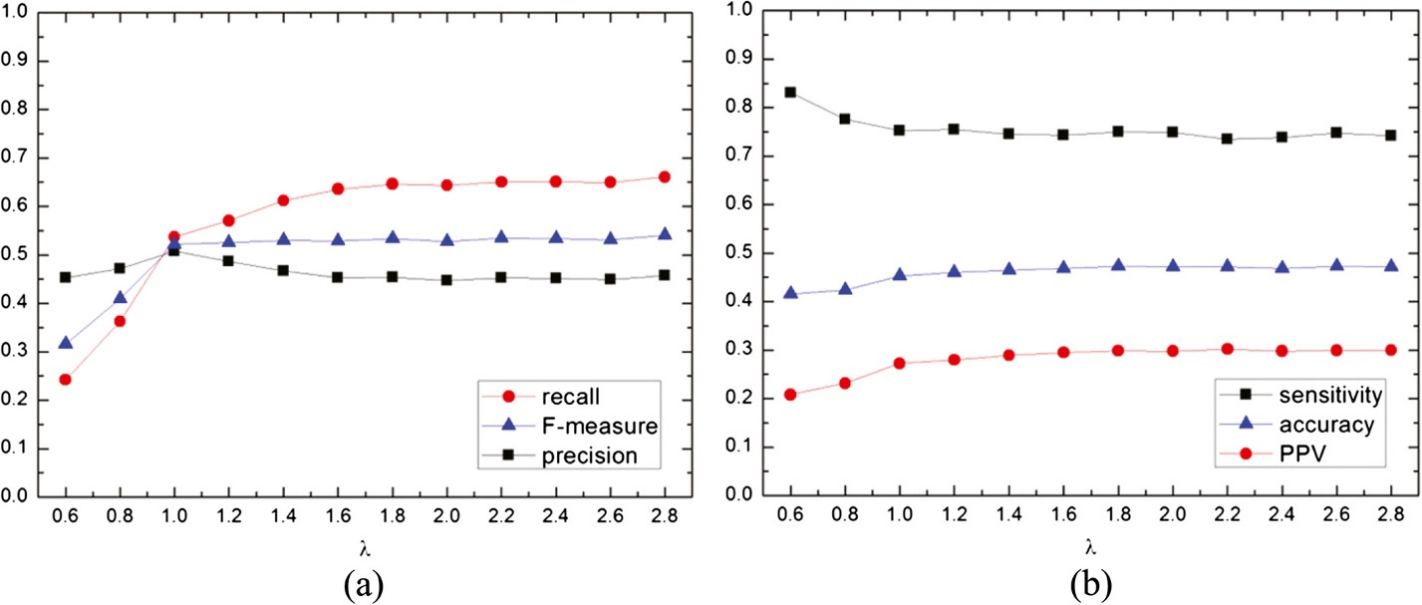
**The effects of merging threshold** ***λ*** **on 6 performance metrics.** **(a)** reveals the relation between the *λ* value and recall, F-measure and precision; and **(b)** displays the relation between the *λ* value and sensitivity, accuracy and PPV.

For the postprocessing process, we employ recall, F-measure, precision, sensitivity, accuracy and PPV metrics to evaluate algorithm performance. Figure 9 gives the effects of merging threshold *λ* on 6 performance metrics. Figure 9(a) demonstrates that the F-measure and recall increase as *λ* increases on the whole range while the precision also increases as *λ* increases at the beginning and decreases after *λ* passes over 1.0. Figure 9(b) shows that the relationship between *λ* and the sensitivity, the accuracy and the PPV. The accuracy and the PPV have the same trend: both values subtly increase as *λ* increases. Conversely, the sensitivity decreases at the beginning, then keep the value low (0.74) after *λ* gets to 1.4. As shown in Figure 9(a) and Figure 9(b), the larger *λ* is, the better F-measure and accuracy seems to be. However, once *λ* is set a larger value, the number of clusters will become too large due to many small clusters. To balance between the scale and size of clusters, the value of *λ* is set to 1.8 in our following experiments.

**Figure 10:**
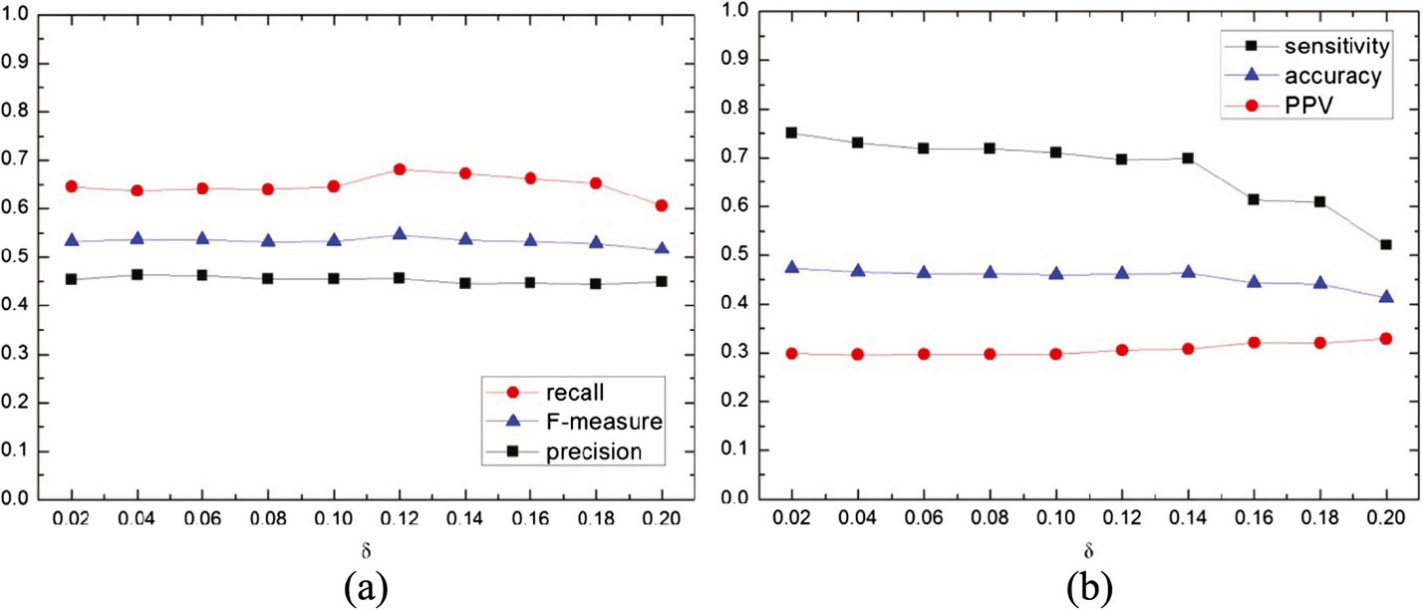
**The effects of filter threshold** ***δ*** **on 6 performance metrics.** **(a)** reveals the relation between the *δ* value and recall, F-measure and precision; and **(b)** displays the relation between the *δ* value and sensitivity, accuracy and PPV.

Figure 10 gives the effects of filter threshold *δ* on 6 performance metrics. As shown in Figure 10(a), the recall and F-measure have a similar trend, namely, their values slowly increase as *δ* increases at the beginning, then gently decrease after *δ* gets to 0.12. However, the rate of change is slightly different for the two metrics where the values of recall have larger changes than those of F-measure. Meanwhile, the precision maintains a relatively stable value around 0.45 though there are two small peaks at *δ*=0.04 and 0.12. Figure 10(b) investigates the relationship between *δ* and PPV, the accuracy and the sensitivity. As *δ* increases, three metrics have different tendencies. In detail, the sensitivity obviously decreases from 0.75 to 0.52, the PPV increases from 0.30 to 0.32 when *δ* locates in [0.02, 0.16], then keeps a larger value (0.32) when *δ*>0.16 while the accuracy holds steady at 0.46 when *δ* varies from 0.02 to 0.14, then slightly decreases from 0.46 to 0.41 when *δ* locates in [0.14, 0.2]. The main reason for these different trends is that only those modules whose similarity is strong enough are merged along with the value of *δ* increasing, thus making the number of clusters to increase and the average size of a cluster to be small. To make a balance, we employ *δ*=0.12 in our algorithm.

Based on similar tests, we have determined the parameter sets for other different data sets, and Table 2 summaries these parameters used in the following experiments.

**Table 2:** **Summary of parameters used in our experiments**

Data sets	Agent random-walk		Multi-agent evolution				Post-processing	
	M	***ε***	R	***p***_***o***_	***p***_***c***_	***p***_***m***_	***λ***	***δ***
Gavin	225	0.25	60	0.5	0.5	0.8	1.8	0.12
Dip	100	0.27	60	0.5	0.5	0.8	0.21	0.04
MIPS	100	0.27	60	0.5	0.5	0.8	0.19	0.05
DIPScere20140703	100	0.29	60	0.5	0.5	0.8	0.6	0.05
DIPHsapi20140703	100	0.28	60	0.5	0.5	0.8	0.6	0.05

From these results, we can give some simple suggestions to preset these parameters. For *M*, a certain size population is necessary for MAE-FMD to obtain good quality solution while keeping a smaller value not to increase the running time. For *ε*, a medium value between [0, 0.6] is recommended. For *R*, a smaller value is favorable to rapidly converge. For *p*_*o*_, *p*_*c*_ and *p*_*m*_, we can set a medium value between [0, 1] to *p*_*o*_ and *p*_*c*_ and a higher value to *p*_*m*_ to save the running time. The two parameters in post-processing depend on different datasets. For the curated databases, such as DIP and MIPS, *λ* and *δ* can be set two smaller values in respective domains, however, two larger values in respective domains have to be employed for the database with more noise (such as Gavin).

### Comparative evaluations

To demonstrate the strengths of the MAE-FMD method, we compared it to the six competing methods: HAMFMD, NACO-FMD, Coach, CFinder, MCL and MCODE in our experiments, where CFinder and MCL run without parameter settings, the only parameter of Coach is the filter threshold *ω* which was set to 0.225, NACO-FMD runs with *α*=1.5, *β*=4 and *δ*=0.3, MCODE adopts the default values for their parameters as provided by its binary executable system, and HAM-FMD uses five different combinations of parameter values (100, 0.5, 2, 4, 286, 0.2, 0.8, 0.1, 0.6), (300, 0.4, 2, 4, 510.8, 0.2, 0.8, 0.3, 0.4), (400, 0.5, 2, 4, 910.8, 0.2, 0.8, 0.1, 0.7), (500, 0.5, 1.5, 5, 1025.2, 0.2, 0.5, 0.1, 0.3) and (400, 0.5, 1.5, 5, 817.2, 0.2, 0.5, 0.1, 0.3) for the parameter set of (m, *ρ*, *α*, *β*, Q, *P*_*o*_, *P*_*c*_, *P*_*m*_, *δ*) on Gavin, DIP and MIPS, respectively.

**Figure 11:**
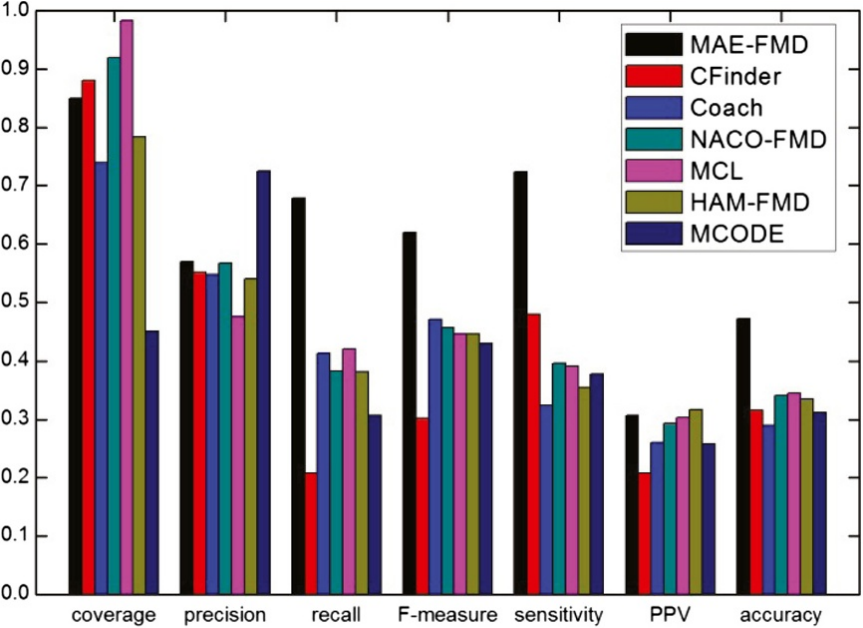
**Comparative results of some methods in terms of various evaluation metrics for Gavin data.**

**Figure 12:**
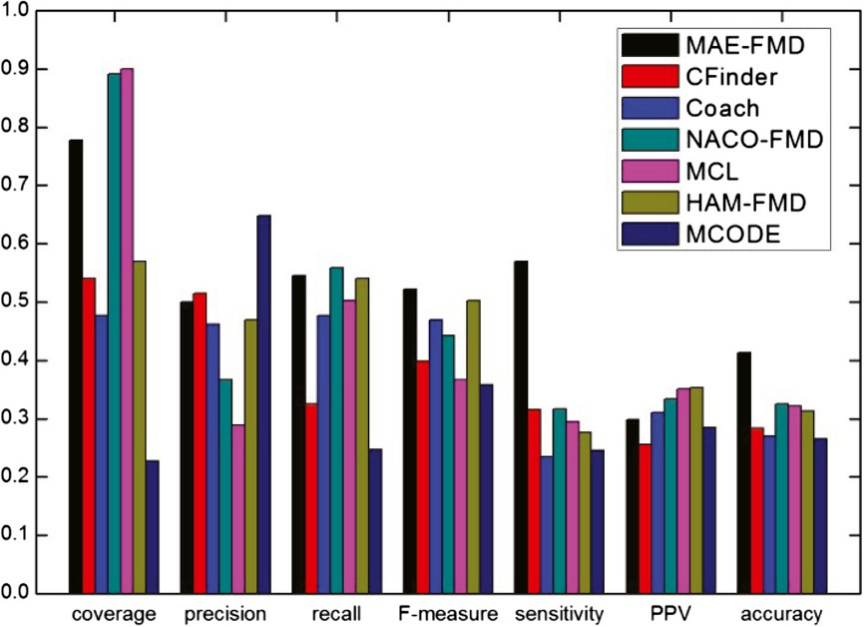
**Comparative results of some methods in terms of various evaluation metrics for DIP data.**

**Figure 13:**
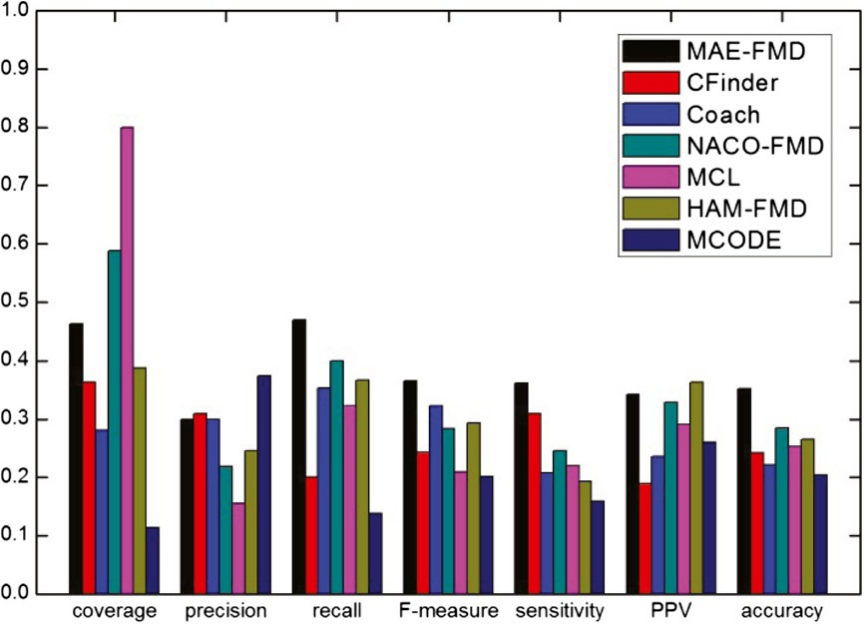
**Comparative results of some methods in terms of various evaluation metrics for MIPS data.**

**Figure 14:**
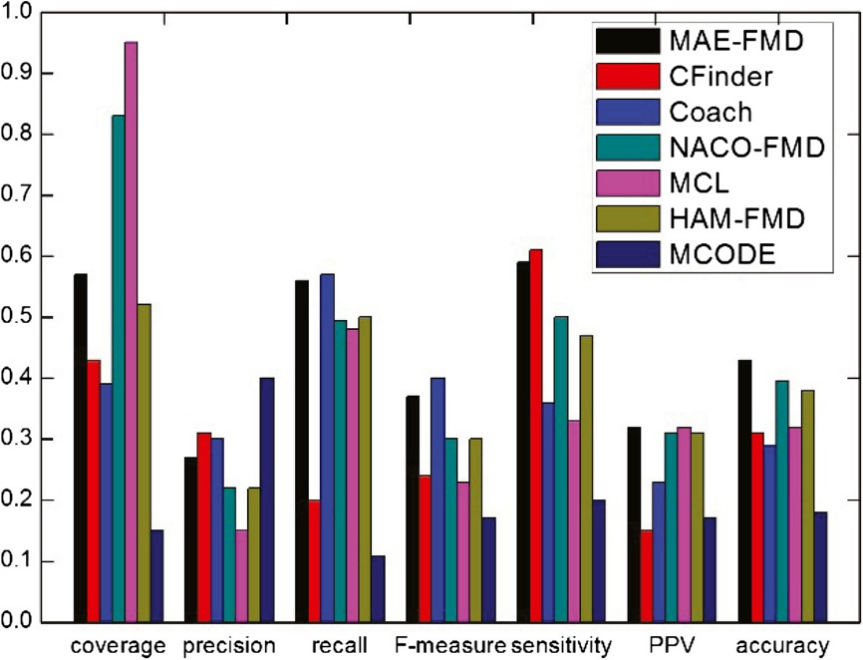
**Comparative results of some methods in terms of various evaluation metrics for DIPScere20140703 data.**

**Figure 15:**
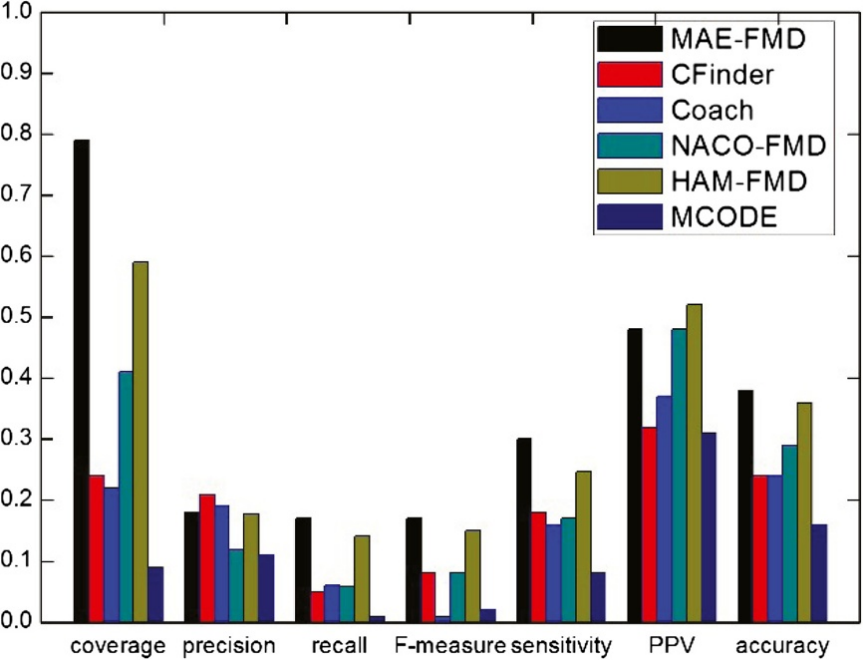
**Comparative results of some methods in terms of various evaluation metrics for DIPHsapi20140703 data.**

The detailed comparative results of the various algorithms on the five different data sets are shown respectively in Table 3, where *"*−*"* denotes an invalid result. For each detection method, we have listed the number of clusters detected (Number of clusters), the average number of proteins in each cluster (size of average module), the number of detected modules which match at least one real module (*N*_*cp*_) and the number of real modules that match at least one detected module (*N*_*cb*_). Taking MAE-FMD on Gavin data as an example, it has detected 193 modules, of which 110 match 224 real modules. Each of 193 detected modules has about 6 proteins in Gavin. These results show that MAE-FMD generates smaller scale clusters on most of data, and MCL doesn’t effectively detect modules when a dataset is largely sparse (i.e. human interaction networks).Figures 11, 12, 13, 14 and 15 show the overall comparison results of these methods in terms of various evaluation metrics, including Coverage, Precision, Recall, F-measure, Sensitivity, PPV and Accuracy for five different data, respectively. From the first panel of these figures, we can conclude that our algorithm archives good performance on the Coverage for all five data sets. For instance, one can easily see that the Coverage of our algorithm is the third highest one among seven algorithms on DIP, MIPS and DIPScere20140703, which is higher than that of other four algorithms and only lower than that of NACO-FMD and MCL. The main reason is that these algorithms adopted different clustering mechanisms which can seriously exert influence on the percentage of proteins clustered into functional modules in a PPI network. Essentially, MAE-FMD, NACO-FMD, HAM-FMD and MCL have two similar characteristics: 1) Three representations of solutions are established on the basis of all nodes of the PPI network. For example, MCL uses a matrix representation of nodes, NACO-FMD employs an ordered sequence of nodes while HAM-FMD and MAE-FMD adopt a connection encoding of nodes; 2) All four algorithms use random clustering mechanisms though specific methods are different. Both characteristics insure that clustering results can include most of nodes in the PPI network. However, MAE-FMD, NACO-FMD and HAM-FMD adopt similar filter operators in post-processing process, thus their coverage values are smaller than that of MCL. Moreover, HAM-FMD combines the random search mechanism used by NACO-FMD with the similar random mechanism used by MAE-FMD, so its coverage value is smaller than those of NACO-FMD and MAE-FMD. Moreover, for the human data (i.e. DIP Hsapi20140703) with large sparsity, our algorithm obtains the best result which shows that MAE-FMD can still keep good coverage performance even when there are seriously sparse connections in the data set.From the second to fourth panels of these figures, we can see that the Precision values of our algorithm are 57%, 50%, 29.9%, 27%, and 18%, respectively. In detail, MAE-FMD obtains the second best result which is only inferior to that of MCODE (72.5%) on Gavin, the third best result which is only inferior to that of CFinder (51.4%, and 30.9%) and MCODE (64.8% and 37.3%) on DIP and MIPS data and that of CFinder (21%) and Coarch (19%) on DIPHsapi20140703 data, and fourth best result which is superior to that of NACO-FMD (22%), MCL (15%) and HAM-FMD (22%) on DIPScere20140703 data. Further, it is easy to observe that our algorithm obtains the best performance on the Recall for Gavin (67.9%), MIPS (46.9%) and DIPHsapi20140703 (17%) data, and is only inferior to that of NACO-FMD on DIP data (less 1.3%) and Coach on DIPScere20140703 data (less 1%). In combination, our algorithm archives the most excellent F-measure on Gavin, DIP, MIPS and DIPHsapi20140703 data, and the second best result on DIPScere20140703 data. That is, our algorithm obtains the highest F-measure value 62.0% with the Gavin data as shown in Figure 11, which is 31.77%, 14.84%, 16.2%, 17.31%, 17.3% and 18.92% higher than that of CFinder, Coach, NACO-FMD, MCL, HAM-FMD and MCODE, 52.2% with the DIP data as shown in Figure 12, which is 12.4%, 5.3%, 7.9%, 15.6%, 2.0% and 16.4% higher than that of CFinder, Coach, NACO-FMD, MCL, HAM-FMD and MCODE, 36.6% with the MIPS data as shown in Figure 13, which is 12.2%, 4.3%, 8.3%, 15.7%, 7.2% and 16.4% higher than that of CFinder, Coach, NACO-FMD, MCL, HAM-FMD and MCODE, 17% with the DIPHsapi20140703 data as shown in Figure 15, which is 9%, 16%, 8.9%, 2% and 15% higher than that of CFinder, Coach, NACO-FMD, HAM-FMD and MCODE, and 37% with the DIPScere20140703 data as shown in Figure 14, which is 13%, 7%, 14%, 7% and 20% higher than that of CFinder, NACO-FMD, MCL, HAM-FMD and MCODE, and only 0.3% lower than that of Coach, respectively.From these figures, we also can observe that MAE-FMD gets the best sensitivity in four data sets (Gavin, DIP, MIPS and DIPHsapi20140703) and the second best result in another data (DIPScere20140703), which indicates the modules detected by our algorithm can cover the real functional modules to a great extent. More specifically, we can see that the sensitivity of our algorithm is 72.4% in Figure 11, which is 24.4%, 40.0%, 32.7%, 33.2%, 36.9% and 34.6% higher than that of CFinder, Coach, NACO-FMD, MCL, HAM-FMD and MCODE algorithms with the Gavin data. Figure 12 shows the sensitivity of our algorithm is 57.0%, which is 25.5%, 33.5%, 25.4%, 27.6%, 29.3% and 32.5% higher than that of CFinder, Coach, NACO-FMD, MCL, HAM-FMD and MCODE algorithms with the DIP data. Figure 13 shows the sensitivity of our algorithm is 36.2%, which is better than that of CFinder (30.9%), Coach (20.7%), NACO-FMD (24.6%), MCL (22%), HAM-FMD (19.3%) and MCODE (15.9%) algorithms with the MIPS data. Figure 15 shows the sensitivity of our algorithm is 30%, which is much better than that of CFinder (18%), Coach (16%), NACO-FMD (17%), HAM-FMD (24.7%) and MCODE (8%) algorithms with the DIPHsapi20140703 data. Though MAE-FMD gets the second best result (59%) on DIPScere20140703 data, it is only inferior to that of CFinder (61%) and also much better than that of Coach (36%), NACO-FMD (50%), MCL (33%), HAM-FMD (47%) and MCODE (20%) algorithms.On Gavin, MIPS, DIPScere20140703 and DIPHsapi20140703 data, MAE-FMD attains the best or the second best PPV value while its PPV performance is not outstanding on DIP data. In detail, the PPV value of MAE-FMD is 30.7% shown in Figure 11, which is 9.9%, 4.7%, 1.4%, 0.4% and 5.0% higher than that of CFinder, Coach, NACO-FMD, MCL and MCODE and is 0.9% lower than that of HAM-FMD. Figure 12 shows the PPV value of MAE-FMD is 29.9%, which is 4.4% and 1.4% higher than the CFinder and MCODE algorithms, and is 1.1%, 3.5%, 5.2% and 5.4% lower than that of Coach, NACO-FMD, MCL and HAM-FMD algorithms with the DIP data. In Figure 13, the PPV value of MAE-FMD is 34.2%, which is 15.3%, 10.6%, 1.2%, 5.1% and 8.2% higher than that of CFinder, Coach, NACO-FMD, MCL and MCODE, and only is 2.1% lower than that of HAM-FMD. The PPV value of MAE-FMD is 32% shown in Figure 14, which is 17%, 9%, 1%, 1% and 15% higher than that of CFinder, Coach, NACO-FMD and MCODE and is equal to that of MCL. In Figure 15, the PPV value of MAE-FMD is 48%, which is equal to that of NACO-FMD, and 16%, 11% and 17% higher than that of CFinder, Coach and MCODE while it is only 4% lower than that of HAM-FMD.Overall, our algorithm achieves the highest Acc on all five tested data due to its balanced effort between Sensitivity and PPV. The Acc value of our algorithm is 47.2% shown in Figure 11, which is 15.6%, 18.2%, 13.1%, 12.8%, 13.7% and 16.1% higher than that of CFinder, Coach, NACO-FMD, MCL, HAM-FMD and MCODE with the Gavin data, respectively. Figure 12 shows the Acc value of our algorithm is 41.3%, which is 12.9%, 14.3%, 8.8%, 9.2%, 10.1% and 14.9% higher than that of CFinder, Coach, NACO-FMD, MCL, HAM-FMD and MCODE with the DIP data, respectively. Figure 13 shows the Acc value of our algorithm is 35.2%, which is 11.0%, 13.1%, 6.8%, 9.9%, 8.7% and 14.9% higher than that of CFinder, Coach, NACO-FMD, MCL, HAM-FMD and MCODE with the MIPS data. Figure 14 shows the Acc value of our algorithm is 43%, which is 12%, 14%, 3.4%, 11%, 5% and 25% higher than that of CFinder, Coach, NACO-FMD, MCL, HAM-FMD and MCODE with the DIPScere20140703 data. Similarly, our algorithm attains 38% on Acc metric, which is 14%, 14%, 9%, 2% and 22% higher than that of CFinder, Coach, NACO-FMD, HAM-FMD and MCODE with the DIPHsapi20140703 data. These experimental results on the Acc performance show that our algorithm is superior to other six algorithms.

**Table 3:** **The results of various algorithms on different data sets**

Data sets	Results	Algorithms						
		MAE-FMD	CFinder	Coach	NACO-FMD	MCL	HAM-FMD	MCODE
	Number of clusters	193	98	325	162	208	163	69
Gavin	Size of average module	6.30	12.91	10.37	8.13	6.76	6.87	9.35
	*N*_*cp*_>0.2	110	54	178	92	99	88	49
	*N*_*cb*_>0.2	224	89	177	164	180	163	86
	Number of clusters	234	173	383	406	500	296	88
DIP	Size of average module	8.40	8.09	5.66	5.57	4.57	4.88	6.52
	*N*_*cp*_>0.2	117	89	177	149	144	139	56
	*N*_*cb*_>0.2	223	139	204	239	215	231	97
	Number of clusters	384	178	488	543	593	449	83
MIPS	Size of average module	5.84	9.29	9.23	4.93	6.16	3.92	6.23
	*N*_*cp*_>0.2	115	55	146	119	92	110	30
	*N*_*cb*_>0.2	197	86	151	173	138	157	55
	Number of clusters	526	204	891	571	968	598	55
DIPScere20140703	Size of average module	5.55	13.03	8.96	7.52	5.04	4.51	14.38
	*N*_*cp*_>0.2	147	65	274	127	148	133	22
	*N*_*cb*_>0.2	242	86	246	212	208	215	47
	Number of clusters	741	202	304	350	—	626	78
DIPHsapi20140703	Size of average module	4.39	5.76	4.57	4.85	—	3.89	5.17
	*N*_*cp*_>0.2	136	43	60	45	—	112	9
	*N*_*cb*_>0.2	136	41	53	47	—	112	9

Table 4 compares the distribution of the p-values of protein modules obtained by 7 different algorithms on DIP data, where the first column gives different types of p-values, the second column lists 7 algorithms, and the third to eighth columns respectively present the number of modules located in the corresponding range while the ninth column shows the ratio of the modules with a p-value and all modules detected for each algorithm. From these results, we can find that MCODE, Coach, and CFinder have the three highest ratios in all the statistics, however, MCODE only obtains the minimum amount of modules while Coach can obtains the maximum amount of modules. The ratio difference of three swarm intelligence algorithms (MAE-FMD, HAM-FMD and NACO-FMD) is not obvious, particularly to MAE-FMD and HAM-FMD. MCL has the worst ratio in three types of p-values. Moreover, it is worth noting that most modules with a p-value are concentrated in the area (1.0e-10, 1.0e-3], and only a few modules fall into the range (0, 1.0e-20] where MAE-FMD has obvious advantages comparing with other algorithms.

**Table 4:** **Distribution comparisons of the p-values of protein modules obtained from different algorithms on DIP**

p-values	Algorithms	Distribution ranges						Ratio
		(0,1 ***.*** 0 ***e*** −30]	(1 ***.*** 0 ***e*** −30,1 ***.*** 0 ***e*** −20]	(1 ***.*** 0 ***e*** −20,1 ***.*** 0 ***e*** −10]	(1 ***.*** 0 ***e*** −10,1 ***.*** 0 ***e*** −3]	(1 ***.*** 0 ***e*** −3,0 ***.*** 05]	(0 ***.*** 05,1]	
	MAE-FMD	4	12	33	100	35	17	0.859
	CFinder	3	4	26	80	32	15	0.925
Biological	Coach	1	2	57	213	74	24	0.966
Process	NACO-FMD	2	5	28	158	96	40	0.810
	MCL	1	5	35	180	110	57	0.733
	HAM-FMD	2	3	27	139	64	25	0.878
	MCODE	0	1	15	55	11	3	0.966
	MAE-FMD	7	14	30	76	28	22	0.756
	CFinder	5	6	18	76	18	10	0.769
Cellular	Coach	2	9	54	187	60	26	0.880
Component	NACO-FMD	2	9	34	137	61	38	0.692
	MCL	3	11	38	144	67	55	0.601
	HAM-FMD	1	6	35	109	51	23	0.760
	MCODE	0	1	21	42	8	7	0.898
	MAE-FMD	4	6	22	72	38	20	0.692
	CFinder	1	3	13	63	36	15	0.757
Molecular	Coach	1	1	20	128	108	45	0.789
Function	NACO-FMD	1	2	19	108	69	47	0.606
	MCL	1	3	22	102	84	50	0.495
	HAM-FMD	0	1	15	94	59	32	0.679
	MCODE	0	0	8	39	13	12	0.818

To further investigate the computational results, 10 protein modules with low p-values and high matching rate predicted by different algorithms using DIP data are respectively presented in Tables 5, 6, 7, 8, 9, 10 and 11. In these tables, the first column is a cluster identifier. The second column indicates the number of proteins in each cluster. The third column gives proteins in the predicted module. The four column lists the corresponding real protein module. The fifth column refers to the matching rate (%) between our predicted module and a real module, which can be computed as *N*_*pm*_/*N*_*pc*_, where *N*_*pm*_ is the number of proteins belonging to the same MIPS module (real module) within the matched module, and *N*_*pc*_ is the number of proteins contained in the matched module. The last three columns show corresponding *p*-values of the predicted module from the view of Biological Process, Cellular Component and Molecular Function. From the column of matching rate, we can see that many of the protein modules detected by the seven algorithms match well with the benchmark modules. The p-values of modules in these tables are very low, which further demonstrates that the modules identified have high statistical significance from three different Gene Ontology categories.

**Table 5:** **Some functional modules predicted by MAE-FMD using DIP data**

ID	Size	Proteins in the predicted module	Real protein module	MR(%)	p-values		
					Biological	Cellular	Molecular
1	5	yor132w yor069w yjl154c yhr012w yjl053w	Retromer module	100	3.13e-09	5.58e-13	4.67e-08
2	13	ykl022c ynl172w yfr036w yhr166c ybl084c ylr127c	Anaphase-promoting	100	4.76e-25	1.82e-31	2.84e-14
		yor249c ygl240w ylr102c ydl008w ydr118w ygl003c					
		ygr225w					
3	10	ymr094w yjr060w ydr318w ygr179c ypl018w ygr140w	Kinetochore module	100	1.70e-07	5.95e-16	2.29e-13
		ymr168c yjr089w ykl049c ykl089w					
4	29	yer148w ygr274c ycr042c ydr448w ydr176w ygr252w	Transcription factor	86.2	2.80e-24	1.48e-25	1.18e-24
		yol148c yel009c ymr236w ygl112c ybr198c ybr081c					
		ydr216w ydr167w ypl254w ypr086w yor023c ycr082w					
		yml114c yml098w ydr392w ydr145w yor194c ykl058w					
		yml015c ypl011c yil129c ymr005w ymr227c					
5	10	yjl203w ydl043c ydl030w ymr240c yor319w ynl286w	Ribonucleo protein	100	4.63e-14	7.74e-19	1.93e-06
		yml049c ymr288w ypl213w yir009w					
6	16	yjl194w yml065w ybr060c yll004w ypr162c ynl261w	DNA replication preinitiation	87.5	3.72e-29	1.46e-30	7.63e-28
		yhr118c ybl023c ylr103c ylr274w yil150c yel032w					
		ybr202w ypr019w ymr216c ygl201c					
7	19	ybl099w ynl315c yjl180c yjr121w ydr322c ylr295c	Mitochondrial proton-transportin	73.7	2.80e-24	1.48e-25	1.18e-24
		ypl271w ydl004w ydr377w yml081c ypr020w ybr039w	ATP synthase				
		yypl078c ydr298c q0085 ykl016c q0080 q0130					
		ydl181w					
8	9	ykr026c ypl237w yor260w yjr007w ydr211w ygr083c	Interacting eIF2 (Sui2/3/4) and	88.9	5.43e-13	2.50e-11	2.89e-14
		ylr291c ypl070w yer025w	eIF2B (Gcd1/2/6/7/Gcn3)				
9	21	yhr041c yer022w ydr308c ybr253w ymr112c yor174w	DNA-directed RNA	90.5	1.55e-18	1.04e-37	2.36e-13
		yol135c ykl028w ykr062w yhr058c yol051w ypr070w	polymerase II				
		ydl005c ybr193c ygl025c ylr071c ybl093c ynl236w					
		ygr104c ycr081w yor140w					
10	6	ylr418c ybr279w yol145c yor123c ygl244w yml010w	Transcription elongation factor	100	7.43e-15	4.11e-11	2.63e-12

**Table 6:** **Some functional modules predicted by CFinder using DIP data**

ID	Size	Proteins in the predicted module	Real protein module	MR(%)	p-values		
					Biological	Cellular	Molecular
1	5	yor132w yjl154c yhr012w yjl053w yor069w	Retromer module	100	3.13e-09	5.58e-13	4.67e-08
2	21	ydl140c ydl108w yor151c yil021w ydr138w yjl140w	DNA-directed RNA	85.7	6.88e-17	3.27e-24	8.45e-19
		ybr154c ypr187w yor210w yml010w ygl070c ykl145w	polymerase II, holoenzyme				
		ydr404c ypl129w ybr279w yor123c yol005c ygl244w					
		yol145c ylr418c ylr384c					
3	20	ydl140c yer165w ymr061w ygl044c ykr002w yer133w	mRNA cleavage factor	90	1.60e-28	7.90e-39	5.71e-13
		ykl059c ykl018w ydr228c ydr195w ynl317w yjr093c					
		yal043c ypr107c ylr277c ydr301w yor179c yor250c					
		ylr115w yol123w					
4	18	yjr121w q0085 q0080 ydl181w ypl078c ybl099w	Mitochondrial proton-transporting	83.3	3.88e-37	3.88e-37	3.88e-37
		ydr298c ypl271w ykl016c ynl315c ybr039w q0130	ATP synthase				
		ydr322c-a yml081c-a ydr377w ydl004w ypr020w ylr295c					
5	13	yjr050w yer013w yal032c ykl095w yll036c ybr188c	Spliceosomal network	92.3	2.08e-18	1.83e-22	1.83e-22
		ygl120c ygr129w ymr213w ydr416w ypr101w ylr117c					
		ypl151c					
6	11	yor361c ymr309c ynl244c ybr079c ypr041w ygr162w	eIF1/eIF3/eIF5 complex	72.7	3.22e-14	5.19e-14	1.34e-18
		ygl049c ymr146c yil071c ydr429c ylr192c					
7	9	ykl052c ykr037c ykr083c ybr156c ypl209c gl061c	Condensed nuclear	88.9	7.78e-17	1.88e-12	9.31e-15
		ygr113w ydr201w ydr016c	chromosome kinetochore				
8	8	yor260w ypl237w ygr083c ykr026c yjr007w yer025w	interacting eIF2 (Sui2/3/4) and	100	5.08e-14	9.27e-12	2.69e-15
		ydr211w ylr291c	and eIF2B (Gcd1/2/6/7/Gcn3)				
9	8	ykl018w ybr175w ybr258c yhr119w yar003w ylr015w	COMPASS	100	4.31e-17	6.70e-21	6.70e-21
		ypl138c ydr469w					
10	6	yel032w ybl023c ylr274w yil150c ybr202w ylr103c	pre-replicative complex	83.3	4.70e-11	4.70e-11	4.12e-10

**Table 7:** **Some functional modules predicted by Coach using DIP data**

ID	Size	Proteins in the predicted module	Real protein module	MR(%)	p-values		
					Biological	Cellular	Molecular
1	5	yor132w yor069w yjl154c yhr012w yjl053w	Retromer module	100	3.13e-09	5.58e-13	4.67e-08
2	11	yor249c ygl240w ydl008w ydr118w ykl022c yfr036w	Anaphase-promoting	100	6.17e-23	1.38e-25	2.81e-16
		yhr166c ybl084c ylr127c ynl172w ylr102c					
3	16	ydr335w ygl092w ykl068w ymr047c ykr082w ydl116w	Nuclear pore	81.3	2.49e-19	5.02e-14	8.81e-13
		ygl172w ylr335w ygr119c ymr308c ygr218w yer165w					
		ydr192c ylr347c yjr042w ynl189w					
4	17	ydr448w ydr176w yol148c yhr041c yer022w ydr392w	Transcription factor	76.5	3.93e-13	3.16e-18	1.88e-06
		ydr308c ypl181w yer148w yel009c ybr198c ybr081c					
		yhr099w ygr274c ymr236w ypl254w ygl112c					
5	8	ydr469w yar003w ybr175w ylr015w ypl138c yhr119w	Chromatin remodeling module	100	4.10e-12	2.99e-13	2.24e-10
		ybr258c ykl018w					
6	8	ycr057c yjl069c ydr449c ylr222c ygr090w ylr409c	Ribonucleoprotein module	87.5	1.09e-11	1.51e-14	7.63e-08
		yjl109c ylr129w					
7	7	ypr110c ynr003c yor116c yor207c ynl113w ykl144c	RNA polymerase III	100	7.97e-15	2.39e-15	2.39e-15
		ypr190c					
8	8	ybl099w yjr121w ydr377w yml081c q0085 ykl016c	Mitochondrial proton-transporting	87.5	5.41e-15	5.41e-15	5.41e-15
		ypl078c ydr298c	and ATP synthase				
9	7	yor260w ykr026c yjr007w ydr211w ygr083c ylr291c	Interacting eIF2 (Sui2/3/4)	100	3.57e-12	2.57e-12	2.89e-13
		ypl237w	and eIF2B (Gcd1/2/6/7/Gcn3)				
10	9	ybl105c yjl002c yel002c yor103c yor085w ydl232w	Oligosaccharyl transferase	88.9	1.48e-13	1.30e-17	1.33e-11
		ygl226c ygl022w ymr149w

**Table 8:** **Some functional modules predicted by NACO-FMD using DIP data**

ID	Size	Proteins in the predicted module	Real protein module	MR(%)	p-values		
					Biological	Cellular	Molecular
1	5	yor132w yor069w yjl154c yhr012w yjl053w	Retromer module	100	3.13e-09	5.58e-13	4.67e-08
2	11	ykl022c yhr166c ybl084c yfr036w ynl172w ylr127c	Anaphase-promoting	100	6.17e-23	1.38e-25	2.81e-16
		yor249c ylr102c ygl240w ydl008w ydr118w					
3	24	yor098c ynl189w yhr129c ygr119c ygl172w yil063c	Nuclear pore	70.8	9.65e-27	4.29e-21	2.36e-19
		ygl092w ydr002w ydr192c ypl174c ylr347c yer009w					
		ykl068w ylr335w ymr047c ymr294w ygl097w ydl116w					
		ykl057c ykr082w ydr488c yjr042w yar002w ypl125w					
4	15	yer148w ydr448w ydr176w yel009c ybr198c ygr274c	Transcription factor TFIIIB	93.3	5.87e-14	4.65e-19	2.36e-10
		ybr081c yol148c ydr167w ymr236w ydr392w ygl112c					
		ypl254w ypl181w ypl011c					
5	9	ybl023c yil150c ylr103c ylr274w ygl201c yel032w	DNA replication preinitiation	77.8	1.86e-14	1.86e-14	9.40e-14
		ybr202w ypr019w ymr216c					
6	9	ynr003c ypr110c ynl113w ydr045c yor116c yor207c	RNA polymerase III	88.9	2.28e-19	4.69e-20	4.69e-20
		ypr190c yhr143w-a ykl144c					
7	12	yll036c ydr416w ybr188c yjr050w yir009w yal032c	Ribonucleo protein	91.7	5.96e-17	3.33e-23	3.33e-23
		ymr213w ygr129w ylr117c ykl095w ypl213w ypr101w					
8	14	ydr228c ypr107c yol123w ymr061w ygl044c yjr093c	mRNA cleavage factor	100	1.23e-30	1.23e-30	1.82e-12
		ykr002w ydr301w ylr277c ynl317w ylr115w yor250c					
		yal043c ykl059c					
9	7	yhr090c yhr099w yor244w yjl081c ypr023c yfl024c	Transcription factor	100	8.08e-09	8.08e-16	2.34e-10
		ynl107w					
10	6	ygl061c ydr201w ykr083c ydr016c ykr037c ykl052c	Kinetochore module	100	2.63e-15	2.63e-15	7.90e-14

**Table 9:** **Some functional modules predicted by MCL using DIP data**

ID	Size	Proteins in the predicted module	Real protein module	MR(%)	p-values		
					Biological	Cellular	Molecular
1	5	yyor132w yor069w yjl154c yhr012w yjl053w	Retromer module	100	3.13e-09	5.58e-13	4.67e-08
2	12	ykl022c ynl172w yfr036w yhr166c ybl084c ylr127c	Anaphase-promoting	100	4.62e-22	2.08e-28	4.13e-15
		yor249c ygl240w ylr102c ydl008w ydr118w ygr225w					
3	8	yar003w ybr175w ydr469w ylr015w yhr119w ypl138c	COMPASS module	100	4.31e-17	4.31e-17	6.70e-21
		ybr258c ykl018w					
4	10	ymr309c ynl244c ypr041w yor361c ymr146c ydr429c	eIF1/eIF3/eIF5 module	80	7.12e-10	2.05e-14	1.51e-13
		yil071c ybr079c ynl062c ylr192c					
5	16	ydr195w ydr228c yor250c ymr061w ypr107c yjr093c	mRNA cleavage factor	93.8	6.79e-26	4.48e-33	1.92e-11
		ykr002w ydr301w ylr115w yal043c ylr277c ynl317w					
		ykl059c yor179c ynl222w ydl094c					
6	7	yjl194w yml065w ybr060c yll004w ypr162c ynl261w	DNA replication preinitiation	100	5.38e-14	4.19e-15	2.64e-12
		yhr118c					
7	13	yor076c ygr158c ydl111c ygr195w ydr280w yol021c	Exosome	100	2.62e-28	4.57e-30	4.36e-02
		yhr069c ynl232w yol142w ycr035c ygr095c yor001w					
		yhr081w					
8	10	ynr003c ypr190c ypr110c ynl113w yor116c ydr045c	RNA polymerase III	100	1.93e-15	4.50e-16	4.50e-16
		yor207c yfr011c ynl248c ykl144c					
9	8	ybl023c ylr103c ylr274w yil150c yel032w ybr202w	DNA replication preinitiation	75	2.93e-12	2.93e-12	3.92e-11
		ymr216c ygl201c					
10	6	ylr418c ybr279w yol145c yor123c ygl244w yml010w	Transcription elongation factor	100	7.43e-15	4.11e-11	2.63e-12

**Table 10:** **Some functional modules predicted by HAM-FMD using DIP data**

ID	Size	Proteins in the predicted module	Real protein module	MR(%)	p-values		
					Biological	Cellular	Molecular
1	5	yor132w yjl154c yhr012w yjl053w yor069w	Retromer module	100	3.13e-09	5.58e-13	4.67e-08
2	12	ybl084c ydl008w ydr118w yfr036w ygl240w ygr225w	Anaphase-promoting	100	4.62e-22	2.08e-28	4.13e-15
		yhr166c ykl022c ylr102c ylr127c ynl172w yor249c					
3	18	yal043c ydr195w ydr228c ydr301w yer032w ygl044c	RNA 3’ end processing factor	72.2	1.44e-33	2.76e-37	6.81e-13
		ygr156w yjr093c ykl018w ykl059c ykr002w ylr115w					
		ylr277c ymr061w ynl317w yor179c yor250c ypr107c					
4	11	q0080 q0130 ybl099w ybr039w ydl004w ydl181w	Mitochondrial proton-transporting	81.8	2.53e-13	2.53e-13	2.53e-13
		ydr322c yjr121w yrl295c ynl315c ypl271w	ATP synthase				
5	10	yar019c ybr127c ydl185w yel051w ygr020c ygr092w	No description	80	1.09e-16	3.49e-16	1.74e-15
		ylr447c ymr054w yor270c yor332w					
6	10	ycr057c ydr449c yer082c ygr090w yjl069c yjl109c	Small-subunit processome	100	4.59e-15	1.46e-18	1.40e-09
		ylr129w ylr222c ylr409c ypl126w					
7	9	ybr156c ydr016c ydr201w ygl061c ygr113w ykl052c	mutLbeta module	88.9	7.78e-17	7.78e-17	9.31e-15
		ykr037c ykr083c ypl209c					
8	8	ydr211w yer025w ygr083c yjr007w ykr026c ylr291c	Interacting eIF2 (Sui2/3/4	100	5.08e-14	9.27e-12	2.69e-15
		yor260w ypl237w	and eIF2B (Gcd1/2/6/7/Gcn3)				
9	7	yar003w ybr175w ybr258c ydr469w yhr119w ylr015w	Transcription factor	100	1.23e-14	1.53e-17	1.53e-17
		ypl138c					
10	7	q0085 ydr298c ydr377w ykl016c yml081c ypl078c	Mitochondrial proton-transporting	85.7	1.64e-12	4.58e-14	1.64e-12
		ypl138c	ATP synthase

**Table 11:** **Some functional modules predicted by MCODE using DIP data**

ID	Size	Proteins in the predicted module	Real protein module	MR(%)	p-values		
					Biological	Cellular	Molecular
1	5	yhr012w yjl053w yjl154c yor069w yor132w	Retromer module	100	3.13e-09	5.58e-13	4.67e-08
2	10	ybl084c ydl008w ydr118w yfr036w ygl240w yhr166c	Anaphase-promoting	100	5.62e-24	8.52e-23	1.29e-14
		ykl022c ylr127c ynl172w yor249c					
3	9	ybl026w ycr077c ydr378c yer112w yer146w yjl124c	Ribonucleo protein	100	1.25e-07	9.18e-14	1.16e-05
		yjr022w ylr438c ynl147w					
4	8	ydl232w yel002c ygl022w ygl226c yjl002c ymr149w	Oligosaccharyl transferase	87.5	9.37e-15	8.19e-19	2.51e-12
		yor085w yor103c					
5	7	ycr002c ydl225w ydr507c yhr107c yjr076c ylr314c	Septin module	71.4	8.31e-12	1.36e-13	2.49e-08
		ynl166c					
6	6	ygr200c yhr187w ylr384c ymr312w ypl086c ypl101w	Elongator holoenzyme	100	7.17e-11	3.11e-16	6.61e-05
7	6	ydr211w ygr083c yjr007w ykr026c ylr291c	Interacting eIF2 (Sui2/3/4)	100	3.14e-10	7.03e-13	3.82e-11
		yor260w	and eIF2B (Gcd1/2/6/7/Gcn3)				
8	6	ybr087w yhr191c yjr068w ymr078c ynl290w yol094c	Ctf18 RFC-like module	100	8.60e-10	2.59e-15	1.70e-08
9	6	q0085 ybl099w ydr377w yjr121w ykl016c	Mitochondrial proton-transporting	83.3	4.59e-10	4.59e-10	4.59e-10
		yml081c	ATP synthase				
10	6	ybr079c ydr429c ylr192c ynl244c yor361c ypr041w	eIF1/eIF3/eIF5 module	100	2.39e-07	2.56e-10	5.52e-11

**Figure 16:**
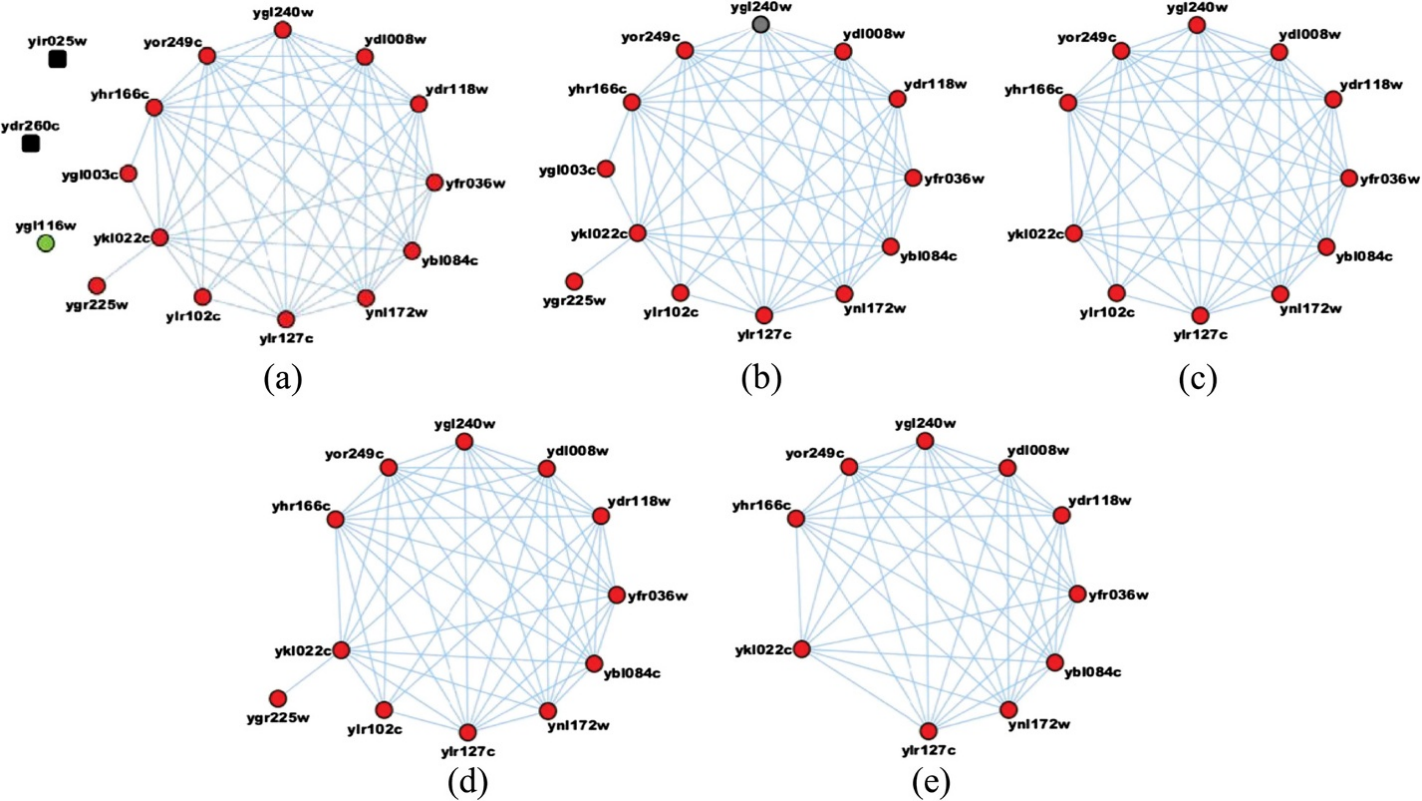
**The anaphase-promoting module detected by various algorithms.** **(a)** Benchmark; **(b)** MAE-FMD algorithms; **(c)** Coach and NACO-FMD algorithms; **(d)** MCL and HAM-FMD algorithms; and **(e)** MCODE algorithm.

To explicitly reveal the results obtained by our algorithm, we take two modules as the examples to explain. For the retromer module, corresponding to the first module in these seven tables, the seven algorithms have obtained the same good performance in terms of p-values and matching rates. That is, the real retromer module is correctly detected by all seven algorithms. Compared to the anaphase-promoting module (corresponding to the second module in Tables 7, 8, 9, 10 and 11) that is respectively detected by the Coach, NACO-FMD, MCL, HAM-FMD and MCODE algorithms, the minimum p-value of our algorithm in Table 5 is 1.82e-31, which is much less than those of the other five algorithms since the minimum p-values of the module predicted by the Coach, NACO-FMD, MCL, HAM-FMD and MCODE algorithms are 1.38e-25, 1.38e-25, 2.08e-28, 2.08e-28 and 5.62e-24, respectively. The real anaphase-promoting module in the benchmark consists of 16 proteins, of which 1 protein (ygl116w) is isolated by other proteins within the same module and 2 proteins (yir025w and ydr260c) don’t exist in DIP data. Thus, the real structure of the anaphase-promoting module including 13 proteins is shown in Figure 16(a). The protein module obtained by our algorithm consists of 13 proteins and succeeds in matching all 13 proteins in the benchmark module (shown in Figure 16(b)). Though the matching rates of Coach, NACO-FMD, MCL, HAM-FMD and MCODE algorithms are also 100%, Coach, NACO-FMD, MCL, HAM-FMD and MCODE only cover 11, 11, 12, 12 and 10 proteins of the real anaphase-promoting module, respectively (shown in Figure 16(c), Figure 16(d) and Figure 16(e)). In addition, CFinder has not obtained the real anaphase-promoting module. Actually, CFinder finds a huge cluster which contains 13 proteins in the real anaphase-promoting module and 49 other proteins. In other words, the example demonstrates that our algorithm can accurately predict protein modules. To show more biological details of Figure 16, Table 12 gives some corresponding messages of the sixteen proteins in anaphase-promoting module.

**Table 12:** **Sixteen proteins in anaphase-promoting module**

ID	Gene name	Protein name	Detail messages (url)
1	yir025w	Anaphase-promoting complex subunit MND2	http://www.uniprot.org/uniprot/P40577
2	ydr260c	Anaphase-promoting complex subunit SWM1	http://www.uniprot.org/uniprot/Q12379
3	ygl116w	APC/C activator protein CDC20	http://www.uniprot.org/uniprot/P26309
4	yor249c	Anaphase-promoting complex subunit 5	http://www.uniprot.org/uniprot/Q08683
5	ylr127c	Anaphase-promoting complex subunit 2	http://www.uniprot.org/uniprot/Q12440
6	ygl240w	Anaphase-promoting complex subunit DOC1	http://www.uniprot.org/uniprot/P53068
7	ylr102c	Anaphase-promoting complex subunit 9	http://www.uniprot.org/uniprot/Q12107
8	ydl008w	Anaphase-promoting complex subunit 11	http://www.uniprot.org/uniprot/Q12157
9	ygr225w	Meiosis-specific APC/C activator protein AMA1	http://www.uniprot.org/uniprot/P50082
10	ydr118w	Anaphase-promoting complex subunit 4	http://www.uniprot.org/uniprot/P0C5L7
11	ykl022c	Anaphase-promoting complex subunit CDC16	http://www.uniprot.org/uniprot/P09798
12	yfr036w	Anaphase-promoting complex subunit CDC26	http://www.uniprot.org/uniprot/P14724
13	ygl003c	APC/C activator protein CDH1 CDC20 homolog 1	http://www.uniprot.org/uniprot/P53197
14	ybl084c	Anaphase-promoting complex subunit CDC27	http://www.uniprot.org/uniprot/P38042
15	yhr166c	Anaphase-promoting complex subunit CDC23	http://www.uniprot.org/uniprot/P16522
16	ynl172w	Anaphase-promoting complex subunit 1	http://www.uniprot.org/uniprot/P53886

Moreover, our algorithm also obtains some new modules on all five data sets. Table 13 lists 5 new modules with lower p-values on the DIP data, which are not previously described or not detected by other six algorithms. This means that MAE-FMD has certain exploratory ability in detection functional modules from a PPI network.

**Table 13:** **Some new functional modules predicted by MAE-FMD algorithm using DIP data**

ID	Size	Proteins in the predicted module	p-values		
			Biological	Cellular	Molecular
1	22	yjr045c yil022w yor232w ybr091c yhr005c ydl217c	2.67e-27	2.04e-21	6.19e-23
		yjl064w yjl143w ynl121c ynl131w yfl016c ynr017w			
		ygr082w ymr203w yel020w ybl030c yml054c yjl054w			
		yor297c ygr181w yjr135w ypl063w			
2	15	ydr382w ylr340w ydl081c ydl130w yel054c	4.66e-12	8.88e-12	1.22e-12
		yol039w ylr287c ylr199c ylr177w yjr125c			
		yor111w ygr034w ymr131c yor063w ygr214w			
3	15	ymr055c yml064c yfr028c yjl076w yjr053w	4.14e-19	1.14e-15	2.26e-12
		ybr211c ygr113w ygl061c ydr016c ydr201w			
		ykr037c ykr083c ykl052c ypl209c ybr156c			
4	13	yll036c ymr213w ydr416w yjr050w ybr188c	2.17e-18	1.91e-22	1.91e-22
		ygr129w ylr117c ykl095w ypr101w yal032c			
		ypl151c ygl120c ydr364c			
5	10	ydr036c ybr251w yhl004w ydr175c ylr009w	1.21e-12	8.40e-14	5.46e-13
		yil093c ygl068w ypl013c ybl038w ynl284c			

In this section, we have performed complete comparisons among MAE-FMD, CFinder, Coach, NACO-FMD, MCL, HAM-FMD and MCODE algorithms in terms of various evaluation metrics (e.g. F-measure, accuracy, p-value etc). These evaluation comparisons from different perspectives show that MAE-FMD is a promising method to effectively identify functional module structures in PPI networks. It should be noted that F-measure and accuracy are two comprehensive evaluation metrics whose values can more objectively reflect the detection quality from different computational views. In light of accuracy, MAE-FMD significantly outperforms CFinder, Coach, NACO-FMD, MCL, HAM-FMD and MCODE on five protein data sets. Based on F-measure, MAE-FMD also outperforms other six algorithms on Gavin, DIP, MIPS and DIPHsapi20140703 data, and is slightly worse than Coach on DIPScere20140703 data. On the other hand, since the p-value of modules is a metric to incarnate the biological significance, the more number of modules we get with lower p-values, the greater significant the application is. Though the number of modules discovered by MAE-FMD is smaller than most of algorithms compared on yeast data, the number of modules with lower p-value discovered by MAE-FMD is no less than those algorithms did. For instance, MAE-FMD detects 234 modules on DIP data which is less than those of HAM-FMD, NACO-FMD, Coarch and MCL, however, the number of modules located in (0, 1.0e-20] is 16, 21 and 10 from three types of p-values, which is much larger than those of other four algorithms. Moreover, MAE-FMD can identify some new modules that were not previously identified by other algorithms, especially for the human data. All these results show MAE-FMD can identify more biological functional modules.

In summary, the outstanding experimental results of MAE-FMD on five different data sets demonstrate that MAE-FMD is robust algorithm whose performances are not dependent on the underlying data.

## Conclusions

To reveal unknown functional ties between proteins and predict functions for unknown proteins, people have remained a great interest in mining functional modules from PPI networks over the past decade. However, how to accurately predict these protein modules through computational methods is still a highly challenging issue. This paper presented a multi-agent evolution approach called MAE-FMD, which can achieve a high accuracy for identifying functional modules in PPI networks. The most significant feature of MAE-FMD is that the algorithm utilizes random search and optimization mechanisms in the solution constructing and evolutionary processes. First, MAE-FMD employs a random-walk model merged topological characteristics with functional information to construct a candidate solution for each agent, which can effectively and reasonably find a feasible solution. And then, it applies some simple evolutionary operators, i.e., competition, crossover, and mutation, to realize information exchange among agents during the evolution process. The competition operator can replace the worst connection information with the better information to improve the winner anent, the crossover operator performs a random search in a solution space by the cooperation between neighborhood agents while the mutation operator carries out local searches with randomness. The experimental results indicate that our algorithm has the characteristics of outstanding recall, F-measure, sensitivity, accuracy and p-value and can obtain some new modules on five benchmark data sets while keeping other competitive performances, so it can be applied to the biological study which requires a higher accuracy. It should be pointed out that the algorithm doesn’t take into account overlapping functional modules based on the current representation and evolution of solutions, and may require longer running time for larger scale PPI networks due to the iterative evolution of the population. Thus, our future work includes investigating some new strategies to further improve the time efficiency and detect overlapping modules in PPI networks.

## Endnote

^a^ Because the underlying protein interaction data used in the paper do not provide temporal and spatial information, we use the concept of functional modules.
